# Supplementary material for: Phylogeography and transmission of M. tuberculosis in Moldova: A prospective genomic analysis
Source: PLoS Med. 2022 Feb 22;19(2):e1003933. doi: 10.1371/journal.pmed.1003933 (PMC8903246; doi:10.1371/journal.pmed.1003933)
Supplement: S1 Appendix — Table A: A summary of the lineages found in mixed M. tuberculosis samples from Moldova, as designated by TB-Profiler. Table B: A summary of the homogeny in drug resistance mutations present in mixed M. tuberculosis samples from Moldova. Table C: In silico drug resistance prediction using TBprofiler and genTB tools. Table D: Allele counts for 9 SNP variants identified in the esxW gene within the study population, showing counts within samples classified as either Beijing strains (all lineage 2.2.1) or as any other lineage. Table E: Demographic associations in cases belonging to large transmission clusters (≥10 cases), identified with patristic distance thresholds of 0.001 and 0.0005. Cases in small clusters (2 to 9 cases) are not included. ORs are calculated using logistic regression and P values by Wald chi-squared test, adjusted for age and sex. Table F: Results of the Coalescent Bayesian Skyline analyses of the 3 large clades with specific resistant mutations using an uncorrelated log normal relaxed clock model. Table G: Complete Coalescent Bayesian Skyline results of the sensitivity analysis using 3 different clock model settings (strict, log normal relaxed, and exponential relaxed) and 3 clock rate estimates of the 3 large clades with specific resistant mutations. The clock rate used log normal distribution. Table H: Pooled Bayesian meta-analysis inference for each exponentiated effect (i.e., ratio of expected SNP distances per specified change in covariate value). Posterior means and 95% quantile-based credible intervals are presented. Fig A: The study flow diagram. Fig B: Distribution of the proportion of MDR-TB by the regions where they were diagnosed. (a) Regions sorted by the proportion of MDR-TB and (b) the total numbers of MDR-TB isolates from high to low. Fig C: (a) A scatterplot showing the pairwise SNP distance (max. 50 SNP differences) plotted against the patristic distance on an M–L phylogeny produced with RAxML between all 2,236 Moldovan isolates wi [file pmed.1003933.s003.pdf]

Supplemental appendix

S1. Study population

All individuals evaluated for tuberculosis, with the exception of those in detention facilities, in the Republic of Moldova in 2018 and 2019 were invited to participate in the study. Consenting individuals with positive sputum cultures were included in this study.

Fig A. The study flow diagram

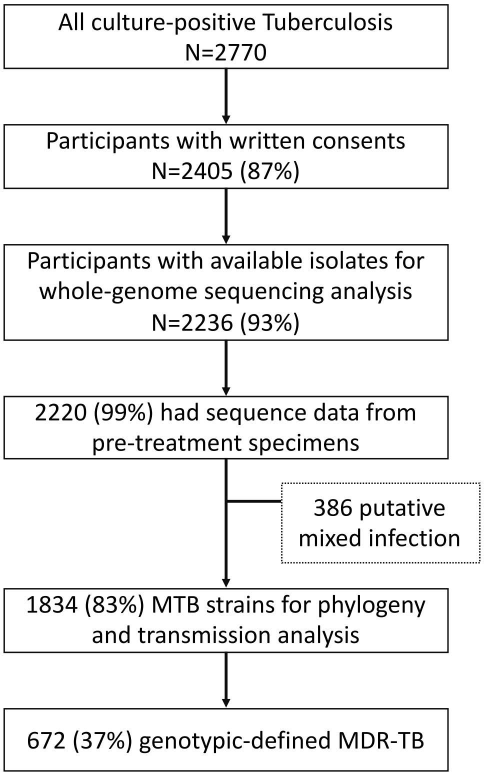

Fig B. Distribution of the proportion of MDR-TB by the regions where they were diagnosed. (a) Regions sorted by the proportion of MDR-TB and (b) the total numbers of MDR-TB isolates from high to low.

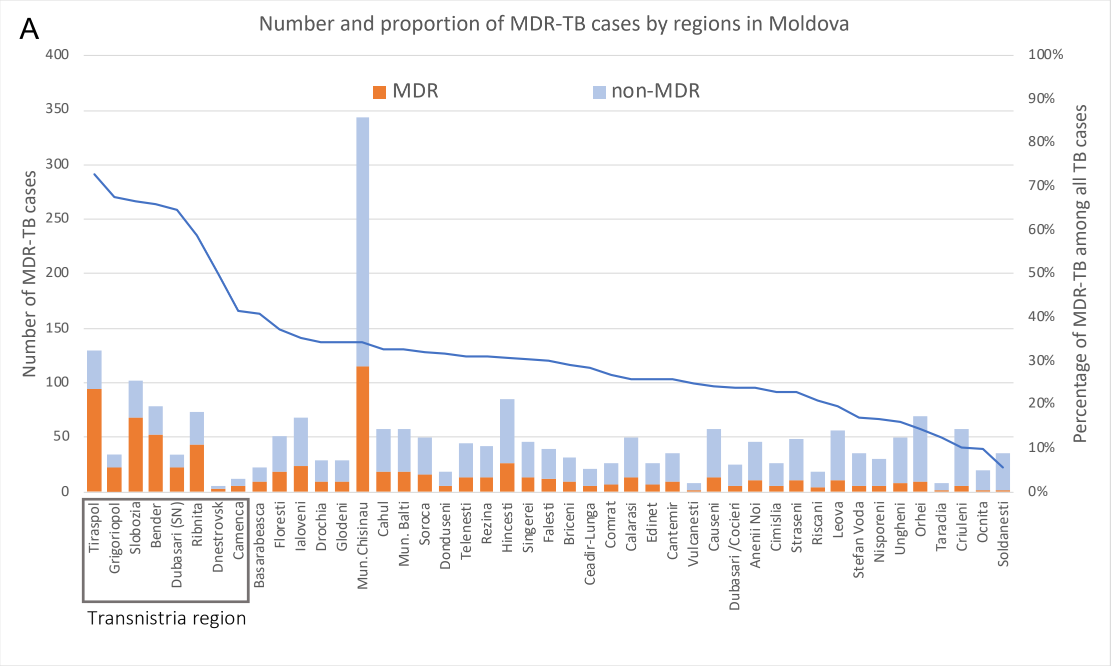

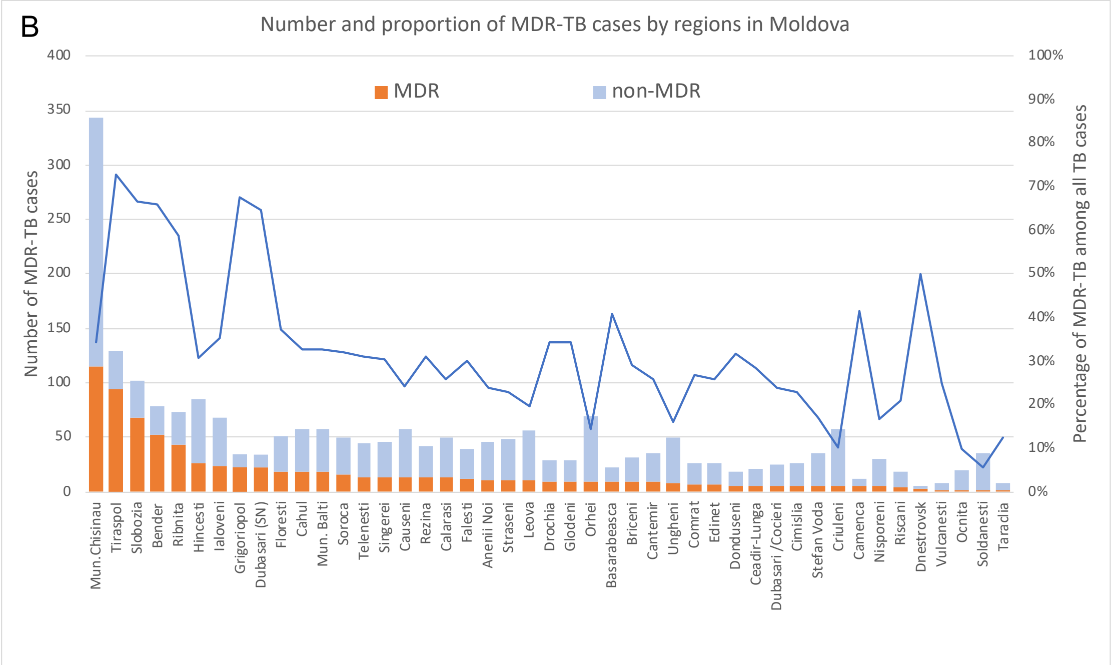

S2. Whole-genome sequencing analysis

Whole-genome DNA preparation methods

Genomic DNA was prepared for whole genome sequencing using the Illumina DNA Prep library preparation kit. Libraries were indexed using the Illumina DNA Prep Unique Dual Indexing system, pooled at equimolar concentrations, and sequenced on an Illumina NextSeq 500

instrument. Up to 285 pooled samples were sequenced on a single NextSeq High Output 300 cycle sequencing kit.

To decrease cost per sample, the Illumina DNA Prep protocol was modified to reduce reagent usage to one fourth of the recommended volumes. Validation of this method showed lower concentration of the final libraries, however no significant loss in genome quality (coverage depth, coverage breadth, sequence duplication levels) was observed compared to full reaction volumes.

Identifying putative mixed infection and related drug-resistant mutations
Preliminary results from an initial genomic analysis of all samples with whole genome sequence data (N = 2236) showed a discordance between the observed pairwise genomic variation (SNP distance) between isolates and their patristic distance on a well-supported maximum-likelihood phylogeny (**Fig C-a**). We aimed to improve the relationship between genomic variation and phylogenetic relatedness by identifying and removing putative mixed infection in our sampled population. We employed the approach detailed in Sobkowiak *et. al.* 2018 for detecting the signal of mixed infection from whole genome sequence data.

Briefly, this method computed the likelihood of a sample containing two or more distinct *Mtb* strains by extracting allele frequencies from the sequencing reads at each position that has been called as a heterogenous site ('0/1') from the GATK variant calling pipeline. A Bayesian clustering approach is then applied to these per-sample heterogenous allele frequencies to determine the likelihood of these frequencies clustering into two or more distinct groups with a mean frequency between 0.2 and 0.8 (indicative of polyclonal infection), or one highly dispersed group (likely clonal variation). The results of this analysis identified 386 possible mixed infections within our sampled population (18.0%), resulting in 1834 non-mixed isolates that were included for the main analysis that showed a strong concordance in the pairwise SNP distance and patristic distance in a maximum-likelihood phylogeny (**Fig C-b**). We also predicted the *in silico* drug resistant mutations and lineage using TB-profiler (Table A and B).

Table A. A summary of the lineages found in mixed *M. tuberculosis* samples from Moldova, as designated by TB-profiler.

**Lineage**	**No. isolates (N = 386)**
lineage4	290
lineage2	68
lineage2 / lineage4 mix	27
lineage3	1

Table B. A summary of the homogeny in drug resistance mutations present in mixed *M. tuberculosis* samples from Moldova.

Drug	Homogeneous sites only	Heterogeneous sites only	Mix of homogeneous and heterogeneous sites
Rifampicin	93	22	4
Isoniazid	119	14	12
Amikacin	13	9	0
Aminoglycosides	13	9	0
Bedaquiline	0	0	0
Capreomycin	14	11	0
Ciprofloxacin	7	11	0
Clofazimine	0	0	0
Cycloserine	2	3	0
Delamanid	2	0	0
Ethambutol	84	31	1
Ethionamide	79	31	0
Fluoroquinolones	7	11	0
Kanamycin	13	9	0
Levofloxacin	7	11	0
Linezolid	0	1	0
Moxifloxacin	7	11	0
Ofloxacin	7	11	0
Para-aminosalicylic acid	6	11	0
Pyrazinamide	31	20	0
Streptomycin	115	32	2

Note: *In silico* drug resistance mutations were predicted using TB-profiler and homogeny of sites (SNPs and INDELs) was carried out by manual inspection of whole genome sequencing assemblies.

We also tested the sensitivity of the *in silico* drug resistance prediction using an online tool-the genTB and compare with the TBprofiler we used in current study. We randomly selected 10% (n=183) of our samples. After excluding one sample with low reads, the prediction of MDR-TB (INH and RIF resistance) was 100% match between the two tools. The comparison results were show in Table C.

Table C *In silico* drug resistance prediction using TBprofiler and genTB tools.

In silico drug resistance with mutations related to	No. of resistance by TBProfiler (n=182)	No. of resistance by genTB online (n=182)
Isoniazid	83	83
Rifampicin	67	67
Pyrazinamide	41	17
Ethambutol	67	59
Streptomycin	77	77
Fluoroquinolones	25	25
Ciprofloxacin	25	23
Levofloxacin	25	22
Ofloxacin	25	23
Ethionamide	52	30
Kanamycin	29	3
Capreomycin	2	2
Amikacin	1	2

Note: https://gentb.hms.harvard.edu/ (on November 17, 2021).

BEAST analysis

Large putative clusters (≥ ten cases) obtained using TreeCluster [1] from a patristic distance threshold of 0.001 substitutions/site were individually analyzed to build timed phylogenetic trees with BEAST2 v2.6.3. [2]. All sequences passed a test for homogeneity of nucleotide composition using IQ-TREE v1.6.12 [3]. Phylogenies were built using a strict molecular clock, calibrated by the tip date of collection, and a fixed clock rate parameter of 1.0×10^{-7} per site per year, equating to 0.44 substitutions per genome per year [4] across all the clusters. We used a coalescent constant population model with a log normal [0,200] prior distribution [4] and a correction for ascertainment bias. We ran the Markov chain Monte Carlo (MCMC) algorithm for 250 million iterations and retained every 25,000-th steps from the posterior. A maximum clade credibility (MCC) tree was generated with the help of TreeAnnotator v2.6.2 [2], with 10% of the chain discarded as burn-in. We used a coalescent Bayesian Skyline model to infer the events of *M. tuberculosis* population expansion to estimate the effective population size change through time in three large clades that were identified in the study population that contained individuals with specific drug resistance mutations. The same strategy was adopted for checking sequence homogeneity composition and optimal model selection (Table C), and each clade was analyzed separately.

Fig C-a. A scatterplot showing the pairwise SNP distance (max. 50 SNP differences) plotted against the patristic distance on a maximum-likelihood phylogeny produced with RAxML between all 2236 Moldovan isolates with whole genome sequence data.

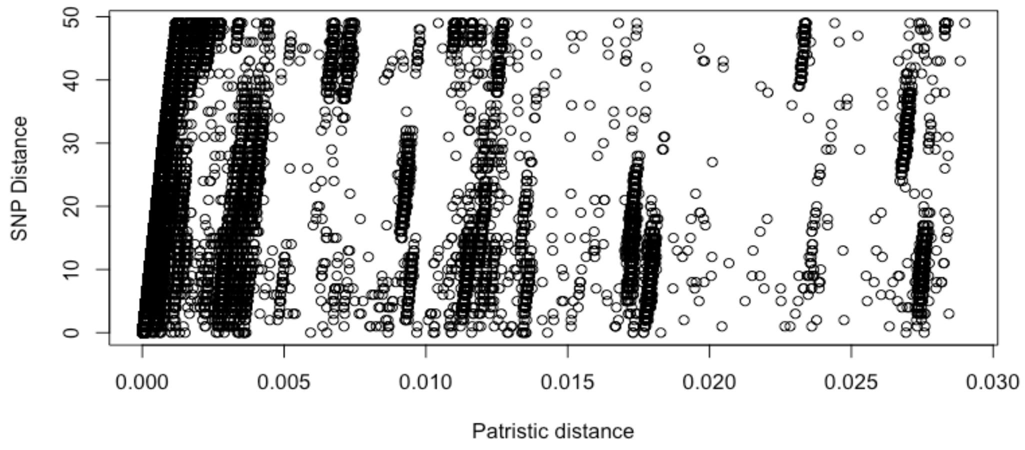

Fig C-b. A scatterplot showing the pairwise SNP distance (max. 50 SNP differences) plotted against the patristic distance on a maximum-likelihood phylogeny produced with RAxML between 1834 non-mixed Moldovan isolates with whole genome sequence data.

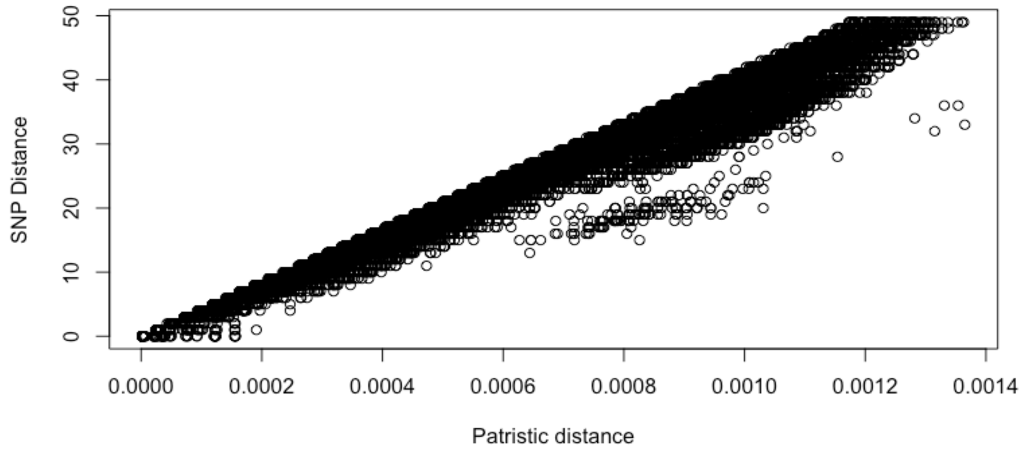

S3. Genomic clustering analysis

Fig D-a. The pairwise SNP distance in 35 large transmission clusters with at least 10 participants involved with the threshold of 0.001. The box plot shows the IQR and median SNP distance of each cluster.

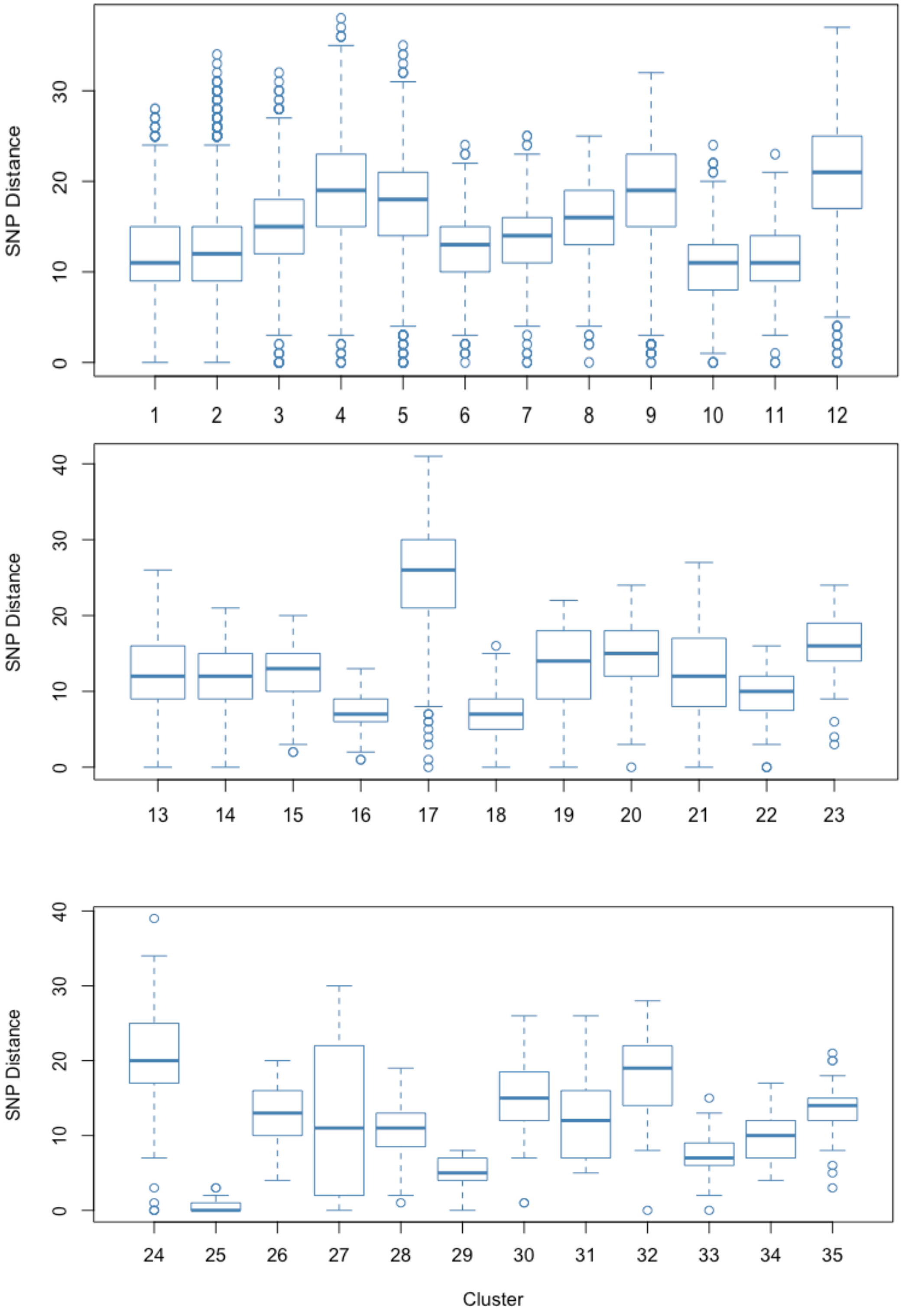

Fig D-b. The pairwise SNP distance in 26 large transmission clusters with at least 10 participants involved with the threshold of 0.0005. The box plot shows the IQR and median SNP distance of each cluster.

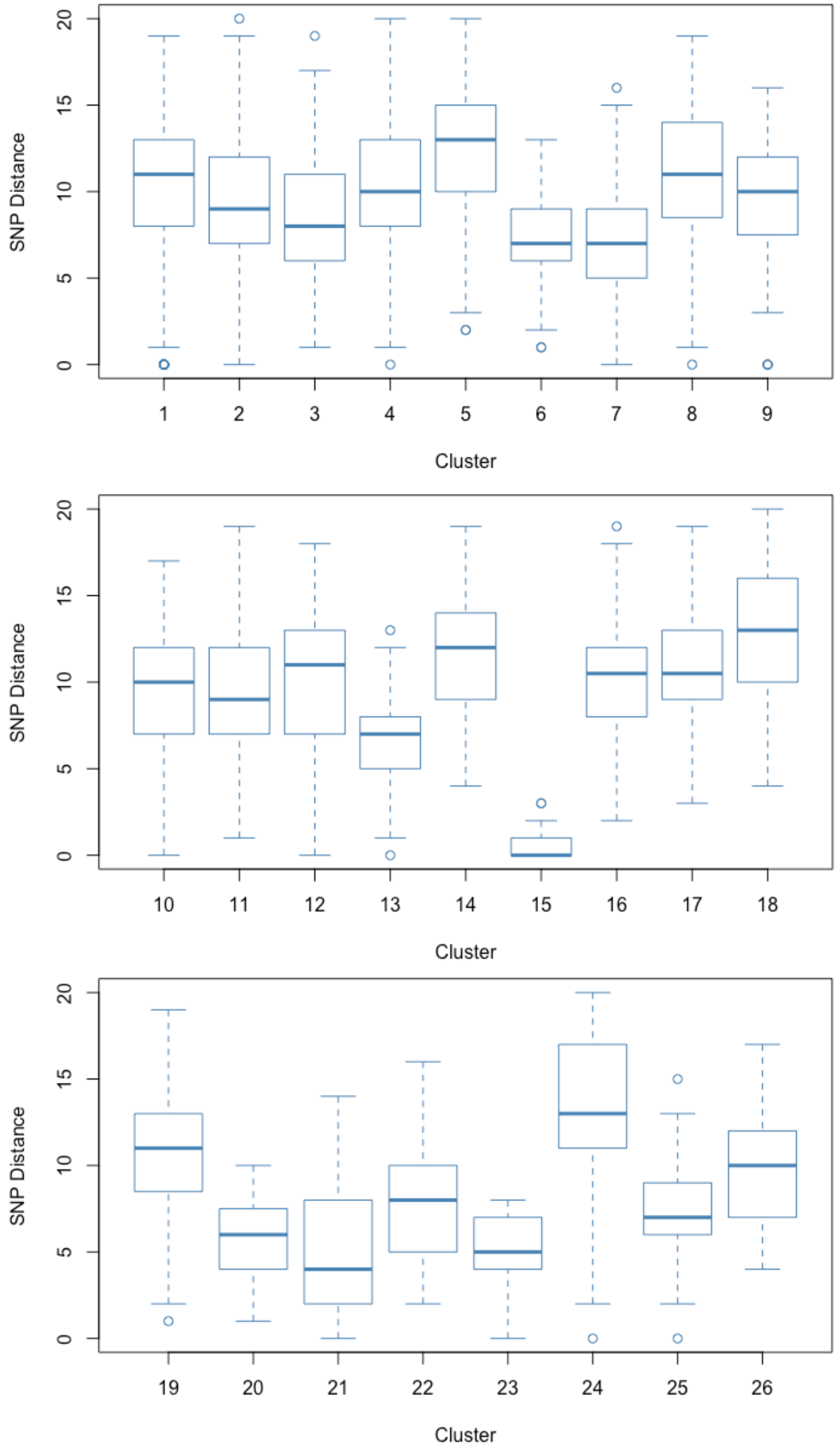

Table D. Allele counts for nine SNP variants identified in the *esxW* gene within the study population, showing counts within samples classified as either Beijing strains (all lineage 2.2.1) or as any other lineage.

			Beijing strain (N = 804)				non-Beijing strains (N = 1030)			
**H37Rv Index**	**Mutation**	**Syn/non-syn**	**Reference allele count**	**Alternative allele count**	**Mixed call**	**Missing call**	**Reference allele count**	**Alternative allele count**	**Mixed call**	**Missing call**
4060334	T-C	S	803	0	0	1	803	213	0	14
4060417	G-A	NS	804	0	0	0	1012	6	0	12
4060418	G-T	S	804	0	0	0	1017	1	0	12
4060420	T-C	NS	804	0	0	0	1001	17	0	12
4060466	G-A	S	804	0	0	0	990	24	4	12
4060469	G-C	S	804	0	0	0	1016	1	1	12
4060472	A-G	S	804	0	0	0	1016	1	1	12
4060545	G-T	S	804	0	0	0	1015	1	0	14
4060562	G-A	S	804	0	0	0	1010	5	0	15
4060588	T-C	NS	0	802	2	0	984	31	1	14

Table E detailed the associated characteristics with the large clusters. We used multivariable logistic regression analysis to calculate the odds ratios (ORs) and 95% CIs for risk factors associated with large genomic clusters, after adjusting for age and sex. Membership in large clusters was found to be significantly associated with age group, previous history of TB treatment and previous imprisonment. Individuals between the ages of 20-29 and 30-29 were more likely to be in large clusters (OR 2.64 and 2.21, *P* value <0.01), as were individuals that either had TB diagnosed as a relapse or treatment failure compared to new cases (OR 1.85 and 2.54, *P* value <0.01), and those that had a history of imprisonment than those that did not (OR 2.57, *P* value <0.01). In addition, individuals in large clusters were more likely to live in Transnistria than the rest of Moldova (OR 3.57, *P* value <0.01) and to reside in an urban center (OR 1.57, *P* value <0.01).

Table E. Demographic associations in cases belonging to large transmission clusters (≥ 10 cases), identified with patristic distance thresholds of 0.001 and 0.0005. Cases in small clusters (2-9 cases) are not included. Odds ratios are calculated using logistic regression and *P* values by Wald chi-squared test, adjusted for age and sex.

		No. in large cluster (cutoff 0.001)	Odds ratio	P value	No. in large cluster (cutoff 0.0005)	Odds ratio	P value
**Total**		1000/1283			404/951		
**Age**							
	<20	24/31	1.60 (0.70 - 4.16)		4/17	0.78 (0.22 - 2.33)	
	20-29	129/151	2.64 (1.62 - 4.47)		56/102	2.94 (1.84 - 4.70)	
	30-39	313/375	2.21 (1.56 - 3.14)		129/259	2.30 (1.63 - 3.27)	
	40-49	242/314	1.45 (1.03 - 2.05)		111/255	1.75 (1.23 - 2.49)	
	50+	268/384	1	**<0.01**	88/291	1	**<0.01**
**Sex**							
	Male	768/980	1		318/732	1	
	Female	232/303	0.79 (0.58 - 1.09)	0.15	86/219	0.70 (0.50 - 0.97)	**0.03**
**TB type**							
	New case	637/856	1		249/641	1	
	Relapse	262/312	1.85 (1.32 - 2.63)		99/214	1.38 (1.00 - 1.90)	

	Treatment failure	76/86	2.54 (1.35 - 5.32)	**<0.01**	39/68	2.09 (1.26 - 3.51)	**<0.01**
**Smear status**							
	Positive	412/540	0.96 (0.76 - 1.28)		169/409	1.19 (0.90 - 1.59)	
	Negative	440/570	1	0.8	159/420	1	0.23
**Drug resistance**							
	Sensitive	456/648	1		166/476	1	
	Drug-resistant	90/129	0.83 (0.55 - 1.29)		43/110	0.86 (0.53 - 1.38)	
	MDR	454/506	3.39 (2.44 - 4.79)	**<0.01**	195/365	2.03 (1.53 - 2.70)	**<0.01**
**Location**							
	Moldova	713/967	1		293/757	1	
	Transnistria	287/316	3.57 (2.40 - 5.49)	**<0.01**	111/194	2.14 (1.55 - 2.97)	**<0.01**

Table E. continued

		**No. in large cluster (0.001)**	**Odds ratio**	**P value**	**No. in large cluster (0.0005)**	**Odds ratio**	**P value**
**Urban dwelling**							
	Yes	427/517	1.57 (1.18 - 2.10)		174/378	1.30 (0.99 - 1.70)	
	No	549/736	1	**<0.01**	214/544	1	0.06
**Homeless**							
	Yes	97/119	1.25 (0.78 - 2.08)		39/92	0.95 (0.61 - 1.48)	
	No	857/1104	1	0.37	342/811	1	0.83
**Monetary assistance**							
	Yes	284/372	1.08 (0.79 - 1.48)		110/276	1.06 (0.78 - 1.44)	
	No	613/780	1	0.62	252/580	1	0.72
**Living conditions**							
	Satisfactory	459/583	1.05 (0.79 - 1.41)		169/423	0.81 (0.60 - 1.07)	

	Unsatisfactory	414/532	1	0.74	173/382	1	0.14
**Occupation**							
	Employed	121/155	1		53/115	1	
	Disabled	95/120	1.16 (0.65 - 2.10)		35/88	0.81 (0.46 - 1.43)	
	Retired	79/123	0.73 (0.41 - 1.27)		26/98	0.63 (0.34 - 1.16)	
	Student	22/27	0.65 (0.23 - 2.16)		3/13	0.19 (0.04 - 0.68)	
	Unemployed	655/825	1.02 (0.66 - 1.54)	0.44	269/606	0.86 (0.57 - 1.29)	0.11
**Education**							
	Primary	338/441	1		134/317	1	
	Secondary	411/537	1.01 (0.74 - 1.36)		154/399	0.86 (0.63 - 1.16)	
	Specialized secondary	169/204	1.52 (1.00 - 2.36)		73/153	1.26 (0.85 - 1.87)	
	Higher education	34/42	1.23 (0.57 - 2.95)		11/27	0.91 (0.39 - 2.03)	
	No education	19/20	5.18 (1.04 - 93.98)	0.15	12/16	3.83 (1.28 - 14.08)	**0.04**
**Previously prisoner**							
	Yes	114/127	2.57 (1.46 - 4.90)		48/84	1.87 (1.18 - 2.99)	
	No	772/1018	1	**<0.01**	302/757	1	**<0.01**

Table F. Results of the Coalescent Bayesian Skyline analyses of the three large clades with specific resistant mutations using an uncorrelated log normal relaxed clock model.

Clades based on DR mutations	# of Taxa	Substitution Model	tMRCA	Clock Rate (SNPs per site per year)
Ural Clade 1	243	TIM with unequal base frequencies	mean: 1984, 95% HPD interval: 1961 - 2003	mean: 2.805×10^{-7}, 95% HPD interval: 1.636×10^{-7} - 3.954×10^{-7}
Beijing Clade 2	102	TVM with equal base frequencies	mean: 2013, 95% HPD interval: 2010 - 2015	mean: 6.248×10^{-7}, 95% HPD interval: 2.445×10^{-7} - 1.087×10^{-6}
Beijing Clade 3	121	TVM with equal base frequencies	mean: 2006, 95% HPD interval: 1999 - 2012	mean: 5.005×10^{-7}, 95% HPD interval: 2.658×10^{-7} - 7.594×10^{-7}

S4. Phylogenetic reconstruction and Bayesian Skyline analysis

Fig E. Tree visualizations for remaining 32 transmission clusters (N ≥ 10 isolates), each showing the location of cases in either the Moldova or Transnistria regions along with resistance/susceptibility to anti-tuberculosis drugs, as identified by *in silico* prediction.

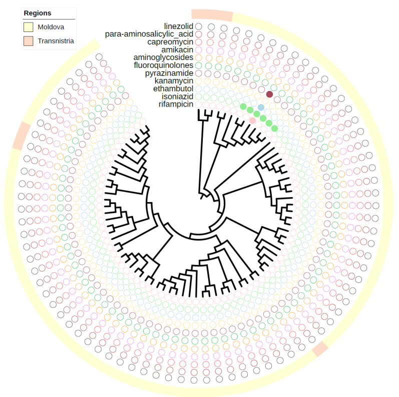

Cluster 3 with 80 isolates

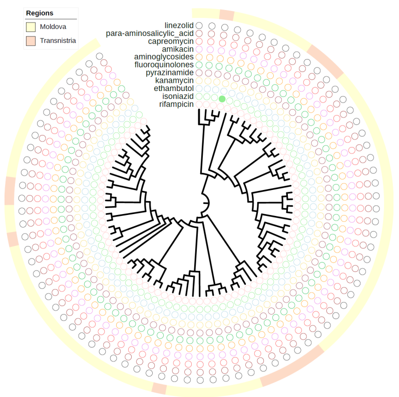

Cluster 4 with 79 isolates

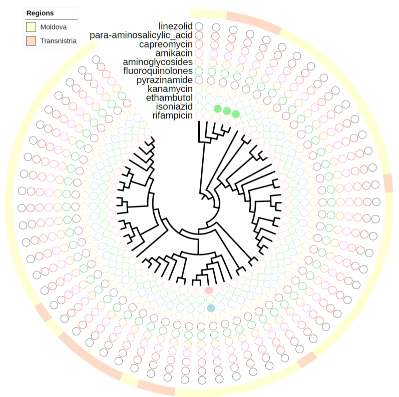

Cluster 5 with 59 isolates

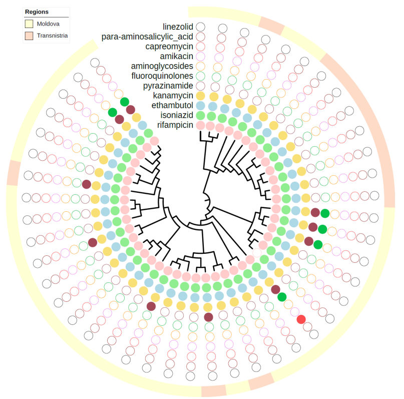

Cluster 6 with 45 isolates

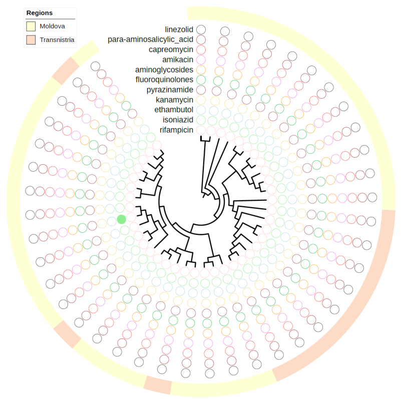

Cluster 8 with 41 isolates

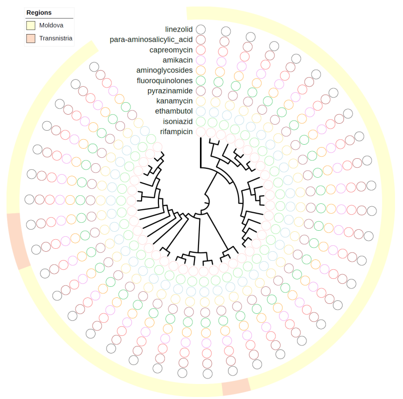

Cluster 9 with 39 isolates

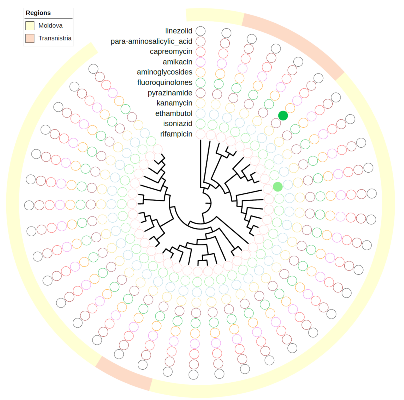

Cluster 10 with 38 isolates

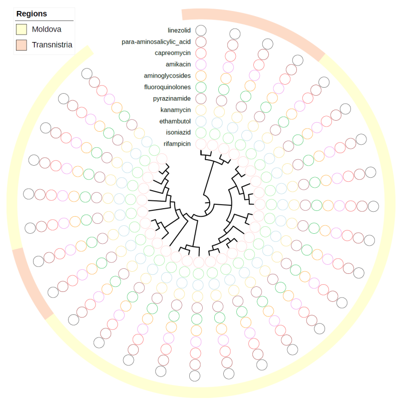

Cluster 11 with 29 isolates

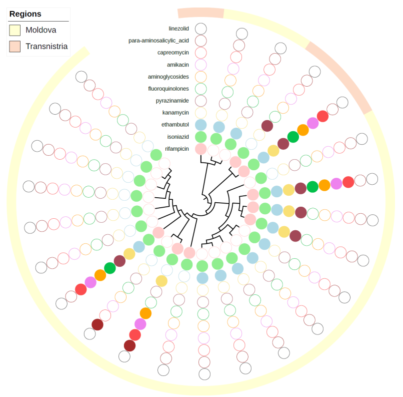

Cluster 12 with 24 isolates

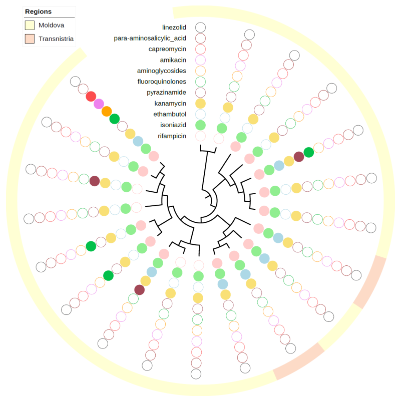

Cluster 13 with 20 isolates

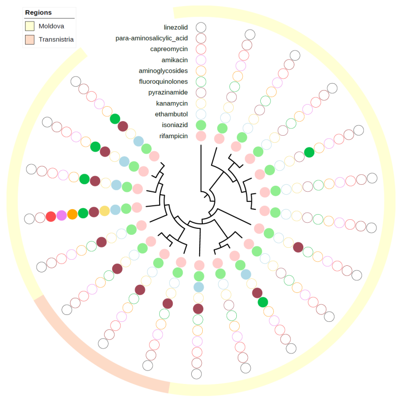

Cluster 14 with 20 isolates

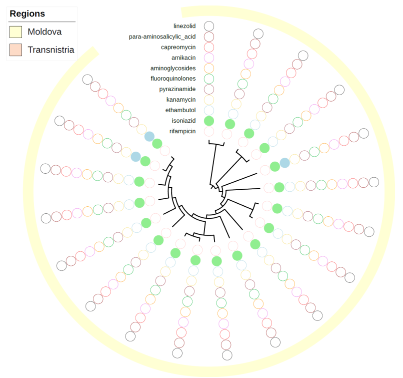

Cluster 15 with 19 isolates

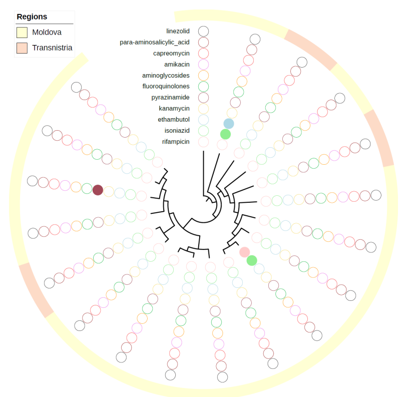

Cluster 16 with 19 isolates

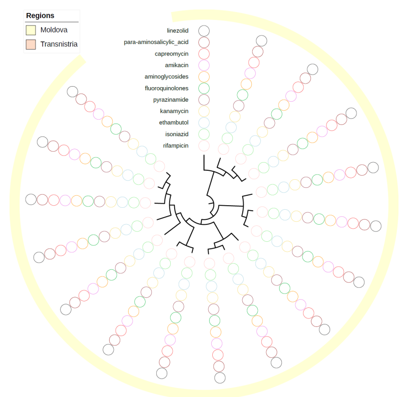

Cluster 17 with 17 isolates

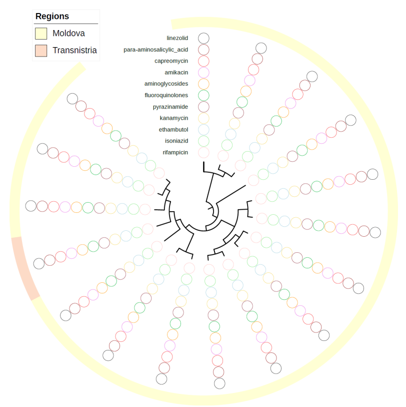

Cluster 18 with 17 isolates

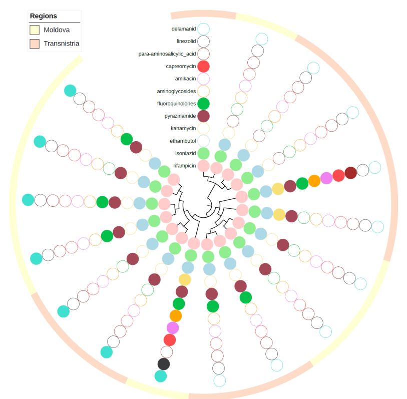

Cluster 19 with 17 isolates

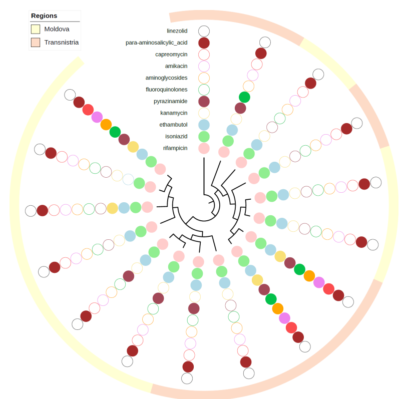

Cluster 20 with 16 isolates

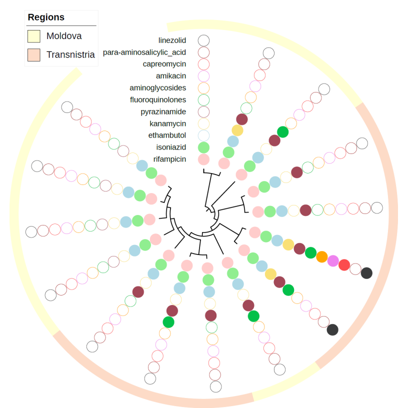

Cluster 21 with 15 isolates

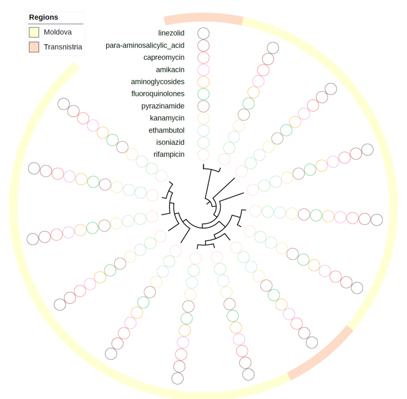

Cluster 22 with 14 isolates

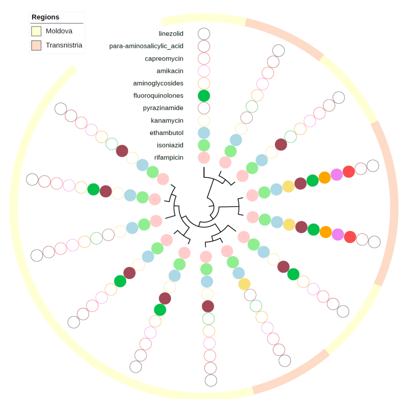

Cluster 23 with 13 isolates

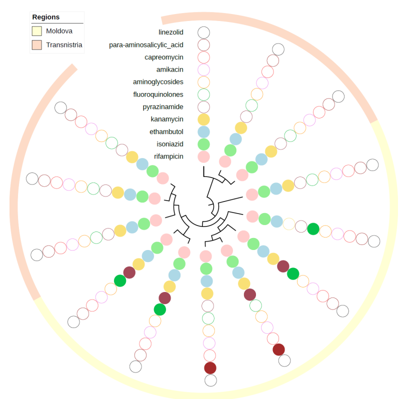

Cluster 24 with 13 isolates

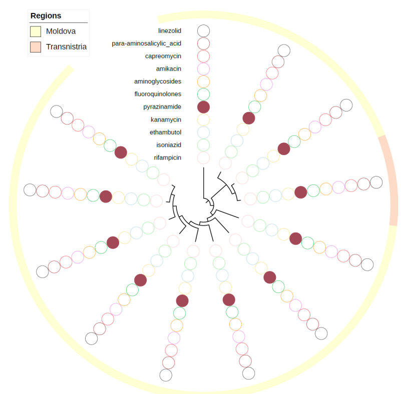

Cluster 25 with 12 isolates

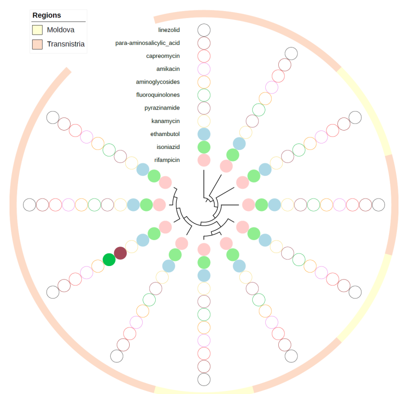

Cluster 26 with 11 isolates

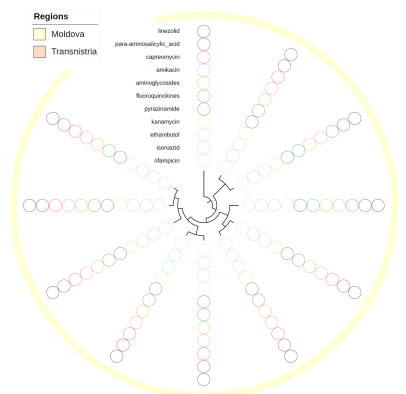

Cluster 27 with 11 isolates

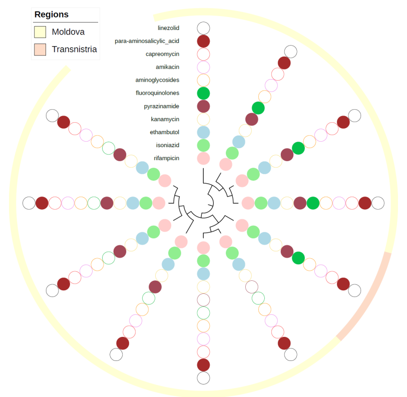

Cluster 28 with 11 isolates

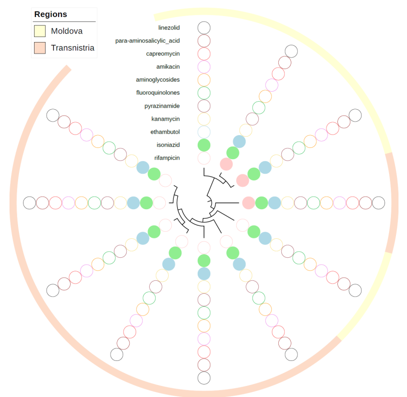

Cluster 29 with 11 isolates

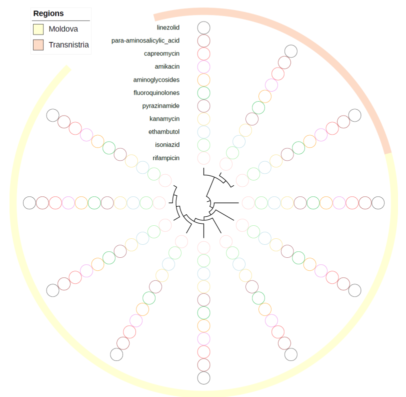

Cluster 30 with 11 isolates

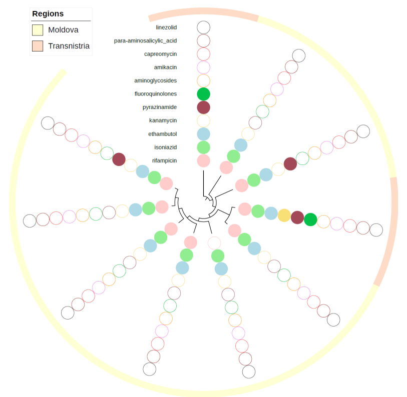

Cluster 31 with 10 isolates

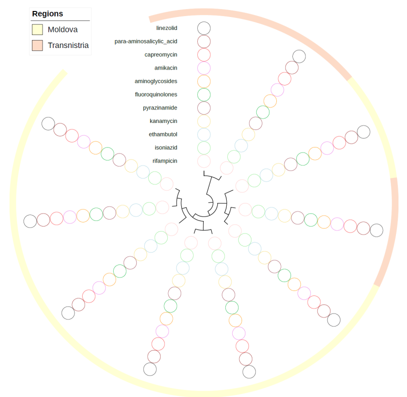

Cluster 32 with 10 isolates

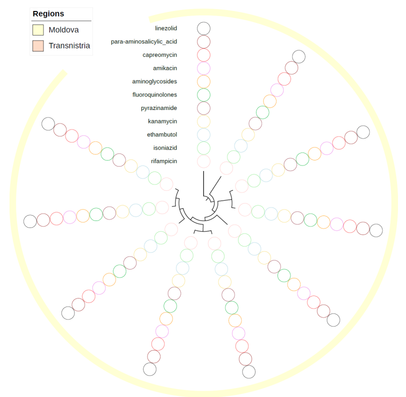

Cluster 33 with 10 isolates

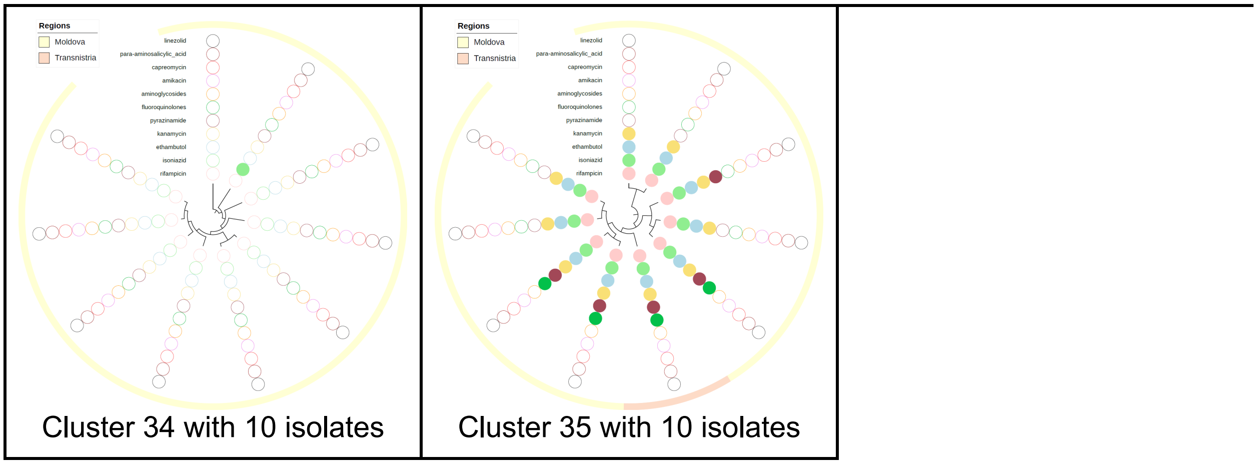

Fig F. Tree visualizations for the 35 transmission clusters (N ≥ 10 isolates), each showing the location of cases in either the Moldova and Transnistria regions along with selected covariates, namely, urban residence, homeless, unsatisfactory living conditions and former prisoner.

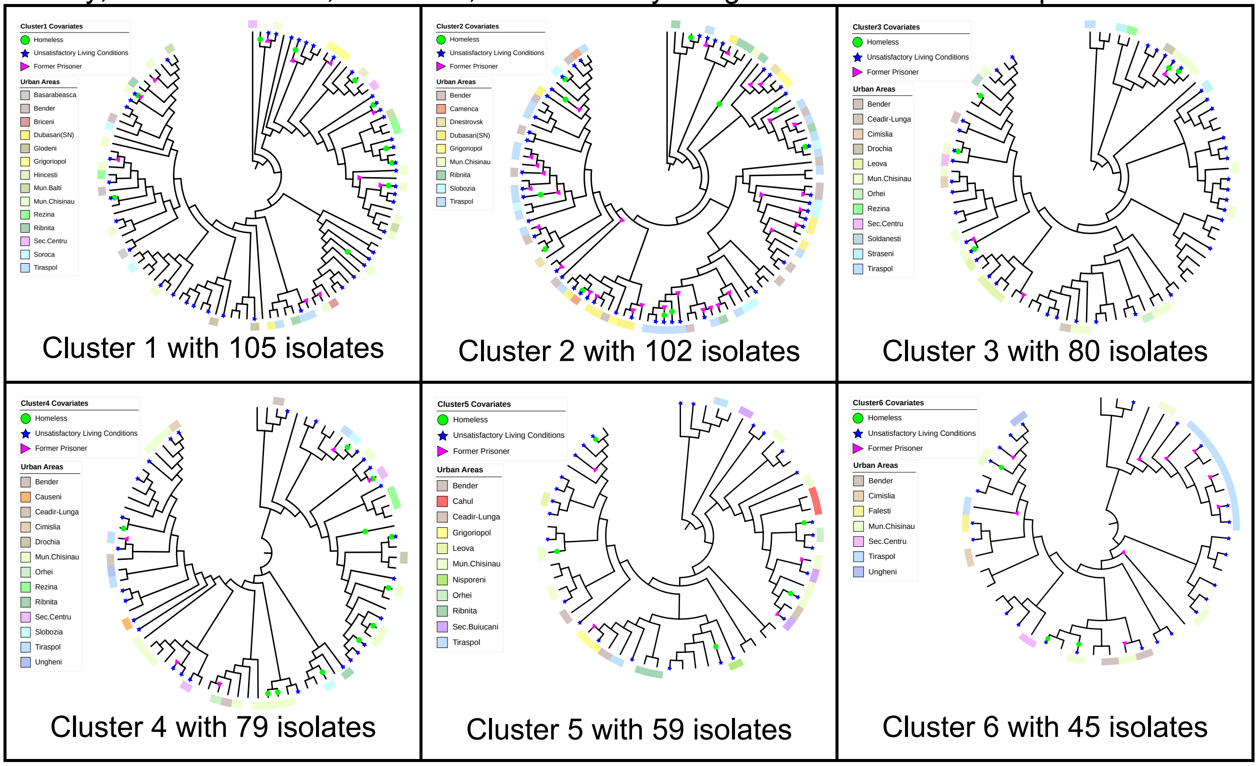

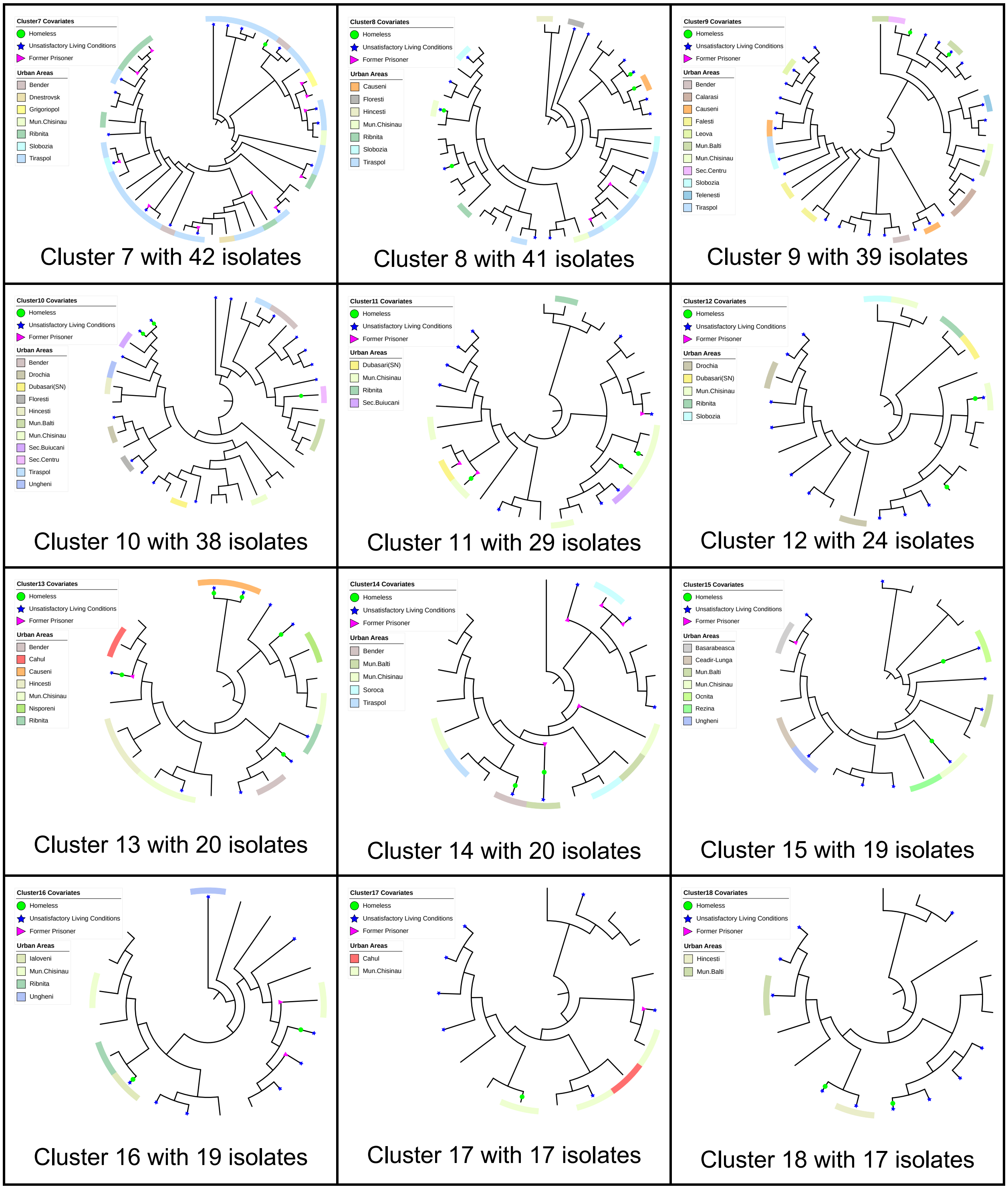

Cluster7 Covariates
Homeless
Unsatisfactory Living Conditions
Former Prisoner
Urban Areas
Bender
Dnestrovsk
Grigoriopol
Mun.Chisinau
Ribnita
Slobozia
Tiraspol
Cluster 7 with 42 isolates
Cluster8 Covariates
Homeless
Unsatisfactory Living Conditions
Former Prisoner
Urban Areas
Causeni
Floresti
Hincesti
Mun.Chisinau
Ribnita
Slobozia
Tiraspol
Cluster 8 with 41 isolates
Cluster9 Covariates
Homeless
Unsatisfactory Living Conditions
Former Prisoner
Urban Areas
Bender
Calarasi
Causeni
Falesti
Leova
Mun.Balti
Mun.Chisinau
Sec.Centru
Slobozia
Telenesti
Tiraspol
Cluster 9 with 39 isolates
Cluster10 Covariates
Homeless
Unsatisfactory Living Conditions
Former Prisoner
Urban Areas
Bender
Drochia
Dubasari(SN)
Floresti
Hincesti
Mun.Balti
Mun.Chisinau
Sec.Buiucani
Sec.Centru
Tiraspol
Ungheni
Cluster 10 with 38 isolates
Cluster11 Covariates
Homeless
Unsatisfactory Living Conditions
Former Prisoner
Urban Areas
Dubasari(SN)
Mun.Chisinau
Ribnita
Sec.Buiucani
Cluster 11 with 29 isolates
Cluster12 Covariates
Homeless
Unsatisfactory Living Conditions
Former Prisoner
Urban Areas
Drochia
Dubasari(SN)
Mun.Chisinau
Ribnita
Slobozia
Cluster 12 with 24 isolates
Cluster13 Covariates
Homeless
Unsatisfactory Living Conditions
Former Prisoner
Urban Areas
Bender
Cahul
Causeni
Hincesti
Mun.Chisinau
Nisporeni
Ribnita
Cluster 13 with 20 isolates
Cluster14 Covariates
Homeless
Unsatisfactory Living Conditions
Former Prisoner
Urban Areas
Bender
Mun.Balti
Mun.Chisinau
Soroca
Tiraspol
Cluster 14 with 20 isolates
Cluster15 Covariates
Homeless
Unsatisfactory Living Conditions
Former Prisoner
Urban Areas
Basarabeasca
Ceadir-Lunga
Mun.Balti
Mun.Chisinau
Ocnita
Rezina
Ungheni
Cluster 15 with 19 isolates
Cluster16 Covariates
Homeless
Unsatisfactory Living Conditions
Former Prisoner
Urban Areas
Ialoveni
Mun.Chisinau
Ribnita
Ungheni
Cluster 16 with 19 isolates
Cluster17 Covariates
Homeless
Unsatisfactory Living Conditions
Former Prisoner
Urban Areas
Cahul
Mun.Chisinau
Cluster 17 with 17 isolates
Cluster18 Covariates
Homeless
Unsatisfactory Living Conditions
Former Prisoner
Urban Areas
Hincesti
Mun.Balti
Cluster 18 with 17 isolates

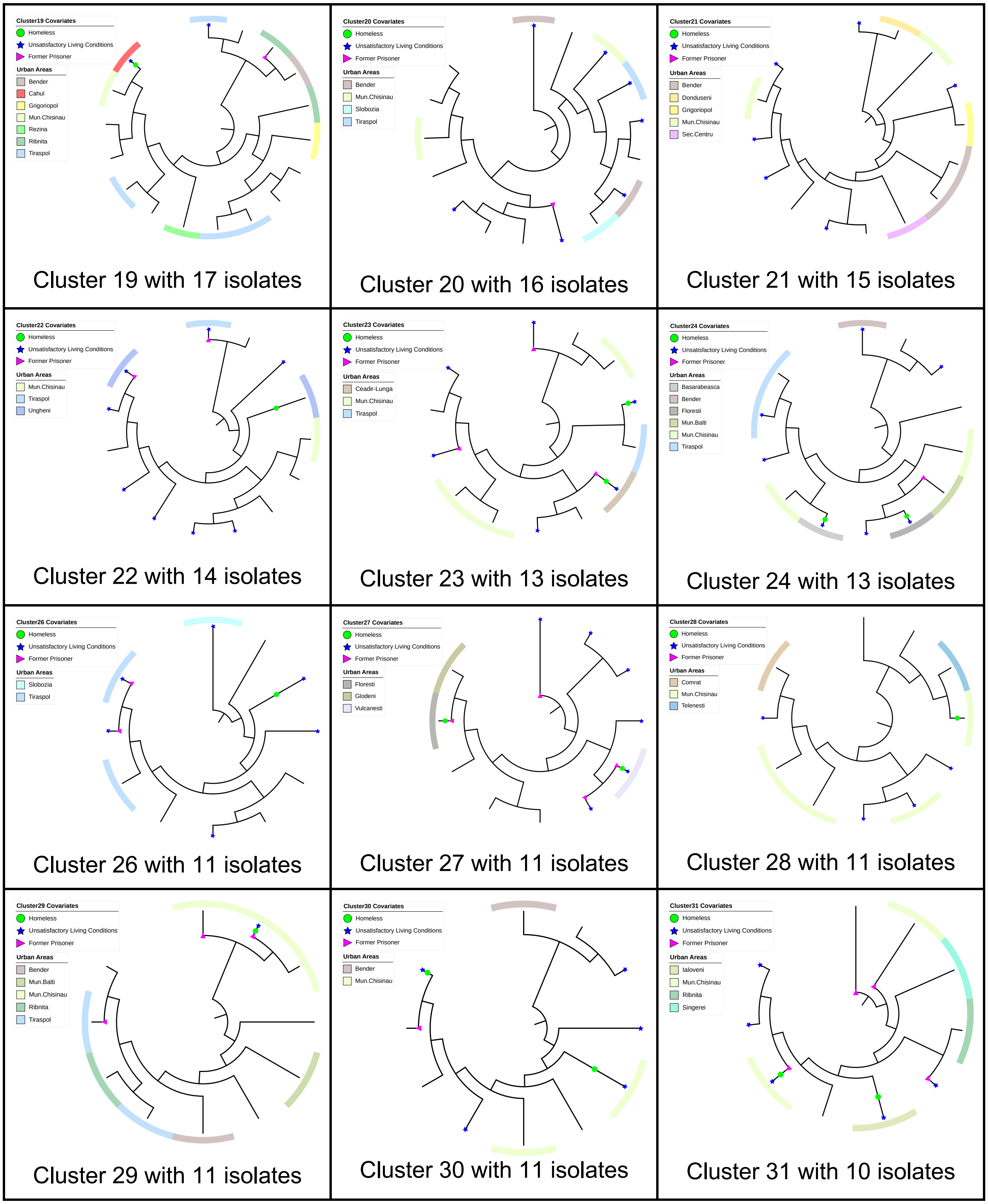

Cluster19 Covariates
Homeless
Unsatisfactory Living Conditions
Former Prisoner
Urban Areas
Bender
Cahul
Grigoriopol
Mun.Chisinau
Rezina
Ribnita
Tiraspol
Cluster 19 with 17 isolates
Cluster20 Covariates
Homeless
Unsatisfactory Living Conditions
Former Prisoner
Urban Areas
Bender
Mun.Chisinau
Slobozia
Tiraspol
Cluster 20 with 16 isolates
Cluster21 Covariates
Homeless
Unsatisfactory Living Conditions
Former Prisoner
Urban Areas
Bender
Donduseni
Grigoriopol
Mun.Chisinau
Sec.Centru
Cluster 21 with 15 isolates
Cluster22 Covariates
Homeless
Unsatisfactory Living Conditions
Former Prisoner
Urban Areas
Mun.Chisinau
Tiraspol
Ungheni
Cluster 22 with 14 isolates
Cluster23 Covariates
Homeless
Unsatisfactory Living Conditions
Former Prisoner
Urban Areas
Ceadir-Lunga
Mun.Chisinau
Tiraspol
Cluster 23 with 13 isolates
Cluster24 Covariates
Homeless
Unsatisfactory Living Conditions
Former Prisoner
Urban Areas
Basarabeasca
Bender
Floresti
Mun.Balti
Mun.Chisinau
Tiraspol
Cluster 24 with 13 isolates
Cluster26 Covariates
Homeless
Unsatisfactory Living Conditions
Former Prisoner
Urban Areas
Slobozia
Tiraspol
Cluster 26 with 11 isolates
Cluster27 Covariates
Homeless
Unsatisfactory Living Conditions
Former Prisoner
Urban Areas
Floresti
Glodeni
Vulcanesti
Cluster 27 with 11 isolates
Cluster28 Covariates
Homeless
Unsatisfactory Living Conditions
Former Prisoner
Urban Areas
Comrat
Mun.Chisinau
Telenesti
Cluster 28 with 11 isolates
Cluster29 Covariates
Homeless
Unsatisfactory Living Conditions
Former Prisoner
Urban Areas
Bender
Mun.Balti
Mun.Chisinau
Ribnita
Tiraspol
Cluster 29 with 11 isolates
Cluster30 Covariates
Homeless
Unsatisfactory Living Conditions
Former Prisoner
Urban Areas
Bender
Mun.Chisinau
Cluster 30 with 11 isolates
Cluster31 Covariates
Homeless
Unsatisfactory Living Conditions
Former Prisoner
Urban Areas
Ialoveni
Mun.Chisinau
Ribnita
Singerei
Cluster 31 with 10 isolates

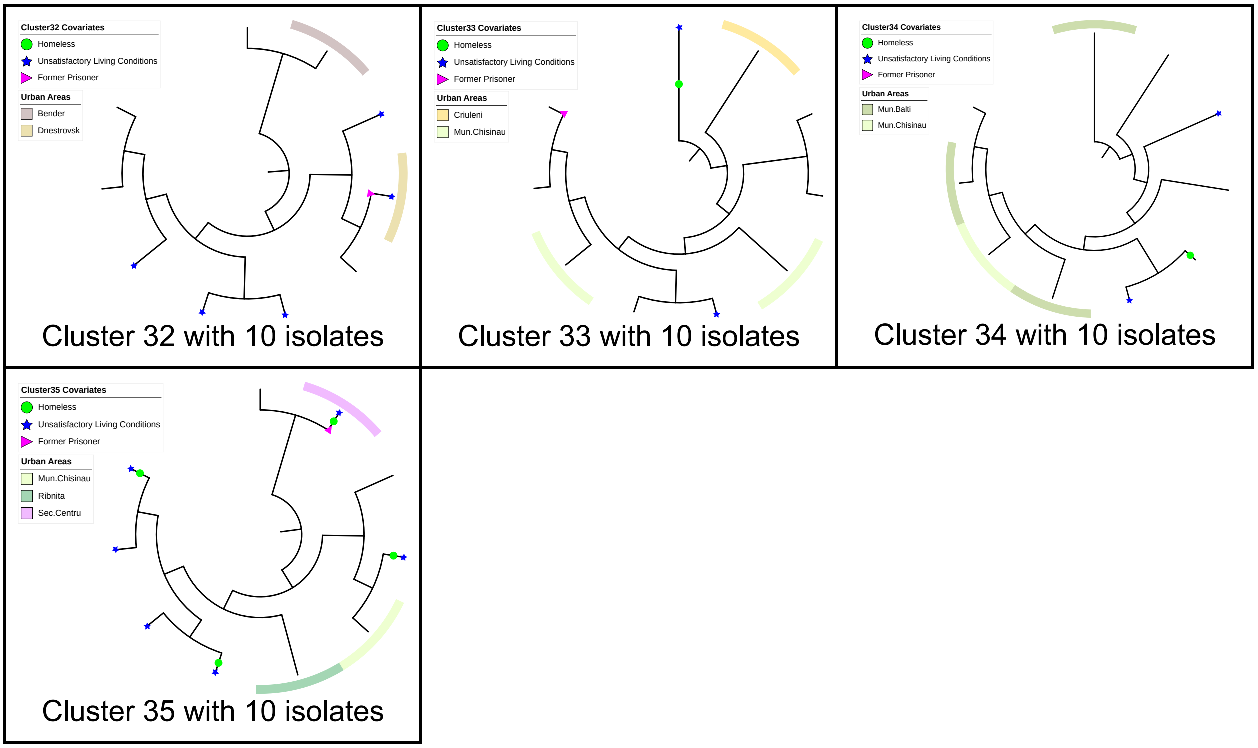
Cluster32 Covariates
Homeless
Unsatisfactory Living Conditions
Former Prisoner
Urban Areas
Bender
Dnestrovsk
Cluster 32 with 10 isolates
Cluster33 Covariates
Homeless
Unsatisfactory Living Conditions
Former Prisoner
Urban Areas
Criuleni
Mun.Chisinau
Cluster 33 with 10 isolates
Cluster34 Covariates
Homeless
Unsatisfactory Living Conditions
Former Prisoner
Urban Areas
Mun.Balti
Mun.Chisinau
Cluster 34 with 10 isolates
Cluster35 Covariates
Homeless
Unsatisfactory Living Conditions
Former Prisoner
Urban Areas
Mun.Chisinau
Ribnita
Sec.Centru
Cluster 35 with 10 isolates

We used a coalescent constant population model with a log normal [0,200] prior distribution. The optimal substitution model for individual clusters was selected on the basis of the Bayesian information criterion (BIC) score using Model-Finder. We adopted the BEAST2 correction for ascertainment bias, defining the number of non-variant A, C, G, and T sites for each cluster, which was manually added to the pre-processed file. We verified chain convergence, as well as good mixing, by calculating effective sample sizes (ESS) (greater than 200) for all parameters across each cluster using Tracer v1.7.1.

Table G. Complete Coalescent Bayesian Skyline results of the sensitivity analysis using three different clock model settings (strict, log normal relaxed and exponential relaxed), and three clock rate estimates of the three large clades with specific resistant mutations. The clock rate used log normal distribution.

					Unfixed clock rate updated through the MCMC iterations		Fixed lineage-specific clock rate based on previous estimates from literature		Fixed clock rate based on prior BEAST analysis of the clades using a constant population	
**Clades based on DR mutations**	**# of Taxa**	**Substitution Model**	**Method**	**Clock Rate Model**	**tMRCA**	**Clock Rate (SNPs per site per year)**	**tMRCA**	**Clock Rate (SNPs per site per year)**	**tMRCA**	**Clock Rate (SNPs per site per year)**
Ural Clade 1	243	TIM with unequal base frequencies	Coalescent Bayesian Skyline	Strict Clock	mean: 1968, 95% HPD interval: 1943 - 1988	mean: 2.123×10^{-7}, 95% HPD interval: 1.263×10^{-7} - 3.018×10^{-7}	mean: 1968, 95% HPD interval: 1944 - 1988	mean: 2.132×10^{-7}, 95% HPD interval: 1.267×10^{-7} - 2.998×10^{-7}	mean: 1967, 95% HPD interval: 1942 - 1988	mean: 2.087×10^{-7}, 95% HPD interval: 1.239×10^{-7} - 2.967×10^{-7}
				Relaxed Clock Exponential	mean: 2002, 95% HPD interval: 1987 - 2013	mean: 4.958×10^{-7}, 95% HPD interval: 2.787×10^{-7} - 7.131×10^{-7}	mean: 2003, 95% HPD interval: 1988 - 2013	mean: 4.971×10^{-7}, 95% HPD interval: 2.901×10^{-7} - 7.043×10^{-7}	mean: 2003, 95% HPD interval: 1989 - 2012	mean: 4.949×10^{-7}, 95% HPD interval: 2.986×10^{-7} - 7.085×10^{-7}
				Relaxed Clock Log Normal	mean: 1984, 95% HPD interval: 1961 - 2004	mean: 2.803×10^{-7}, 95% HPD interval: 1.626×10^{-7} - 3.998×10^{-7}	mean: 1984, 95% HPD interval: 1961 - 2003	mean: 2.805×10^{-7}, 95% HPD interval: 1.636×10^{-7} - 3.954×10^{-7}	mean: 1984, 95% HPD interval: 1961 - 2003	mean: 2.796×10^{-7}, 95% HPD interval: 1.624×10^{-7} - 3.996×10^{-7}
Beijing Clade 2	102	TVM with equal base frequencies	Coalescent Bayesian Skyline	Strict Clock	mean: 2008, 95% HPD interval:	mean: 2.88×10^{-7}, 95% HPD interval: 1.605×10^{-7}	mean: 2007, 95% HPD interval:	mean: 2.847×10^{-7}, 95% HPD interval: 1.581×10^{-7}	mean: 2008, 95% HPD interval:	mean: 2.886×10^{-7}, 95% HPD interval: 1.642×10^{-7}

					2001 - 2013	- 4.243 x 10^{-7}	2001 - 2013	- 4.178 x 10^{-7}	2001 - 2013	- 4.184 x 10^{-7}
				Relaxed Clock Exponential	mean: 2013, 95% HPD interval: 2011 - 2015	mean: 7.502 x 10^{-7}, 95% HPD interval: 3.76 x 10^{-7} - 1.155 x 10^{-6}	mean: 2013, 95% HPD interval: 2011 - 2015	mean: 7.37 x 10^{-7}, 95% HPD interval: 3.757 x 10^{-7} - 1.134 x 10^{-6}	mean: 2013, 95% HPD interval: 2011 - 2015	mean: 7.307 x 10^{-7}, 95% HPD interval: 3.626 x 10^{-7} - 1.14 x 10^{-6}
				Relaxed Clock Log Normal	mean: 2013, 95% HPD interval: 2010 - 2015	mean: 6.311 x 10^{-7}, 95% HPD interval: 2.449 x 10^{-7} - 1.081 x 10^{-6}	mean: 2013, 95% HPD interval: 2010 - 2015	mean: 6.248 x 10^{-7}, 95% HPD interval: 2.445 x 10^{-7} - 1.087 x 10^{-6}	mean: 2013, 95% HPD interval: 2010 - 2015	mean: 6.415 x 10^{-7}, 95% HPD interval: 2.652 x 10^{-7} - 1.084 x 10^{-6}
Beijing Clade 3	121	TVM with equal base frequencies	Coalescent Bayesian Skyline	Strict Clock	mean: 2002, 95% HPD interval: 1994 - 2008	mean: 3.402 x 10^{-7}, 95% HPD interval: 2.031 x 10^{-7} - 4.827 x 10^{-7}	mean: 2002, 95% HPD interval: 1994 - 2008	mean: 3.368 x 10^{-7}, 95% HPD interval: 1.986 x 10^{-7} - 4.776 x 10^{-7}	mean: 2002, 95% HPD interval: 1994 - 2008	mean: 3.403 x 10^{-7}, 95% HPD interval: 2.033 x 10^{-7} - 4.842 x 10^{-7}
				Relaxed Clock Exponential	mean: 2009, 95% HPD interval: 2002 - 2014	mean: 8.96 x 10^{-7}, 95% HPD interval: 4.796 x 10^{-7} - 1.333 x 10^{-6}	mean: 2009, 95% HPD interval: 2003 - 2014	mean: 8.891 x 10^{-7}, 95% HPD interval: 4.812 x 10^{-7} - 1.331 x 10^{-6}	mean: 2009, 95% HPD interval: 2002 - 2014	mean: 8.974 x 10^{-7}, 95% HPD interval: 4.929 x 10^{-7} - 1.327 x 10^{-6}

				Relaxed Clock Log Normal	mean: 2006, 95% HPD interval: 1999 - 2012	mean: 5.037×10^{-7}, 95% HPD interval: 2.743×10^{-7} - 7.487×10^{-7}	mean: 2006, 95% HPD interval: 1999 - 2012	mean: 5.005×10^{-7}, 95% HPD interval: 2.658×10^{-7} - 7.594×10^{-7}	mean: 2006, 95% HPD interval: 1999 - 2012	mean: 5.031×10^{-7}, 95% HPD interval: 2.721×10^{-7} - 7.635×10^{-7}

Bayesian Skyline plot analysis
We used a coalescent Bayesian Skyline model to infer the events of *M. tuberculosis* population expansion to estimate the effective population size change through time in three large clades that were identified in the study population that contained individuals with specific drug resistance mutations. We used an uncorrelated log normal relaxed molecular clock and ran the MCMC algorithm for 250 million iterations, retaining every 25,000-th step from posterior, with the resulting log files analyzed using Tracer v1.7.1 for MCMC convergence and ESS values of greater than 200 across all parameters.

We also conducted a sensitivity analysis using three different clock model settings (strict, log normal relaxed, and exponential relaxed), and three clock rate estimates (**Fig G**): 1) an unfixed clock rate updated through the MCMC iterations, 2) a fixed lineage-specific clock rate based on previous estimates from the literature [4], and 3) a fixed clock rate based on prior BEAST analysis of the clades using a constant population.

Fig G. Coalescent Bayesian Skyline plots of the sensitivity analysis using three different clock model settings (strict, log normal relaxed and exponential relaxed), and three clock rate estimates of the three large clades with specific resistant mutations.

Clades based on DR mutations	Clock Rate Model	Unfixed clock rate updated through the MCMC iterations	Fixed lineage-specific clock rate based on previous estimates from literature	Fixed clock rate based on prior BEAST analysis of the clades using a constant population
Ural Clade 1	Strict Clock	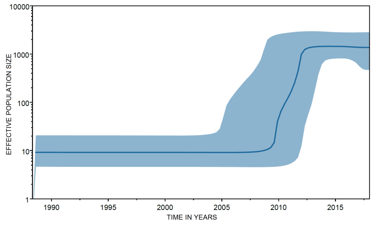	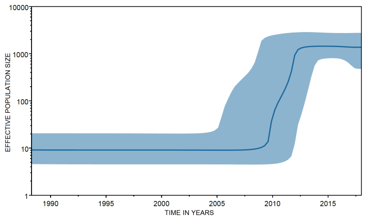	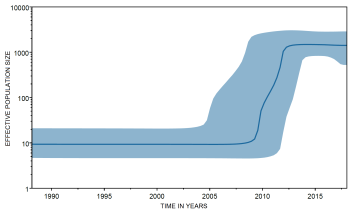
	Exponential Relaxed Clock	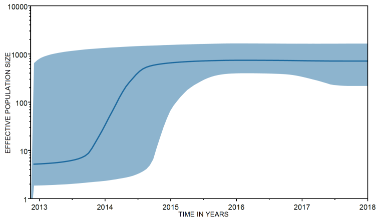	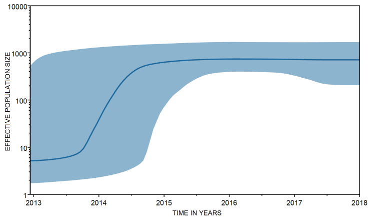	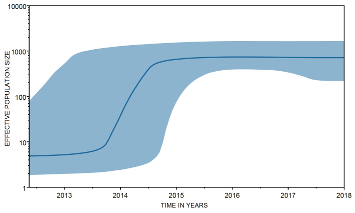
	Log Normal Relaxed Clock	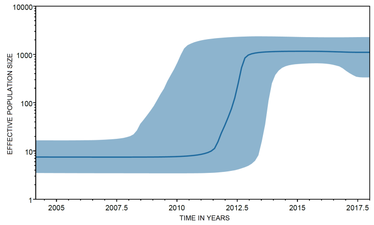	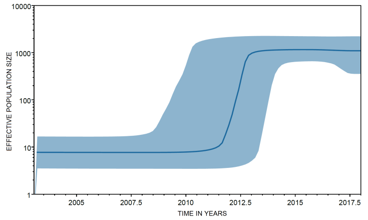	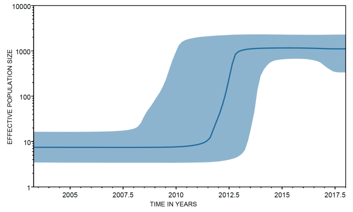

Beijing Clade 2	Strict Clock	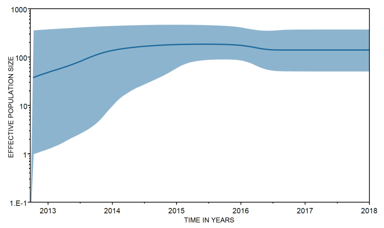	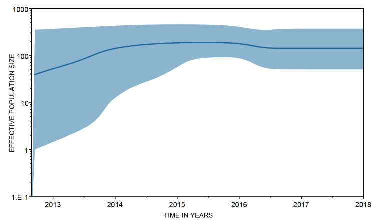	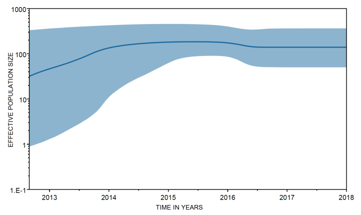
	Exponential Relaxed Clock	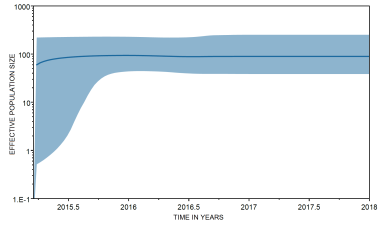	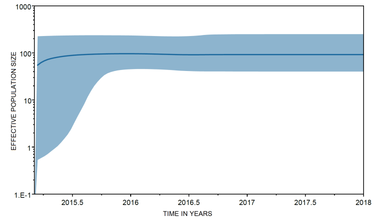	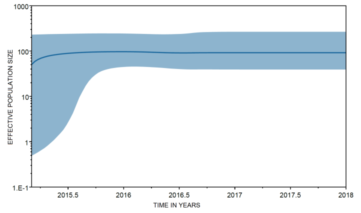
	Log Normal Relaxed Clock	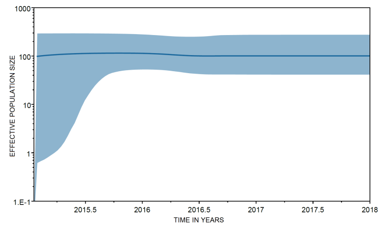	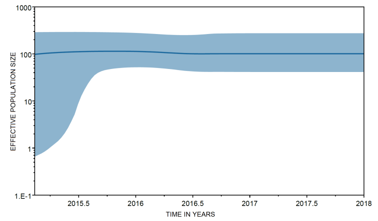	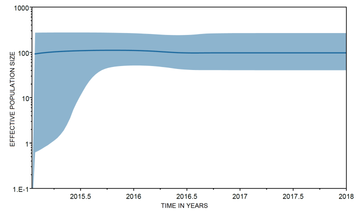
Beijing Clade 3	Strict Clock	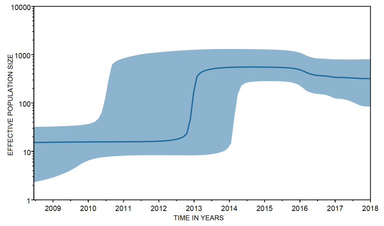	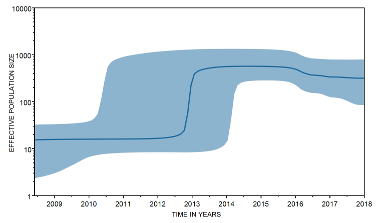	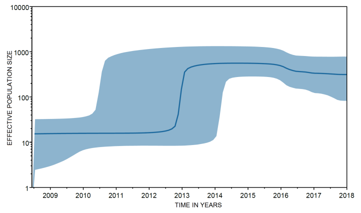
	Exponential Relaxed Clock	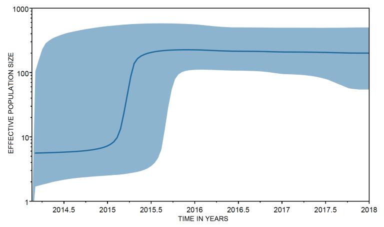	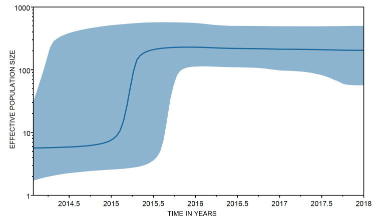	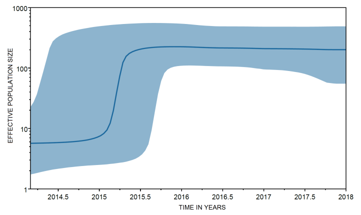
	Log Normal Relaxed Clock	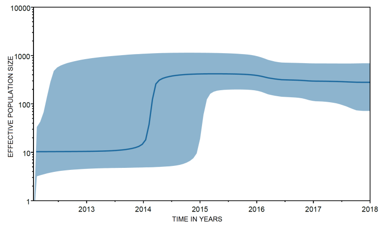	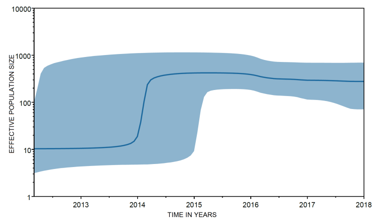	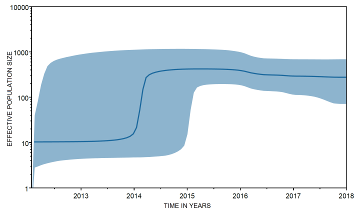

S5. Spatial/genomic distance analysis

For the spatial and genomic distance model, we performed a sensitivity analysis by modeling the SNP distance (instead of the patristic distance) between a pair of cases as a function of geographic distance and other covariates using a negative binomial regression framework similar to that described in the *Spatial/genetic distance analysis* subsection of the main text. These analyses were conducted using the “SNP” function in the R package “GenePair” (https://github.com/warrenjl/GenePair).

In **Table H** we show the pooled RR inference for each of the effects. With respect to geographic distance, we see that two cases in the same locality have a 23% reduction in expected SNP distance compared to cases in different localities (estimate: 0.77 (0.6, 0.88)).
For these cases in different locality, as the distance between the localities increases by 50 kilometers, the SNP distance between the pair increases by about 3% (estimate: 1.03 (1.02, 1.05)). For every half-year increase in the separation between dates of diagnosis for a pair, the SNP distance increased by 1% (estimate: 1.01 (1.00, 1.02)). The only other significant effect is sex, where pairs comprised of two females tend to have larger SNP distances than male only (estimate: 0.91, (0.83, 1.00)) pairs.

Table H. Pooled Bayesian meta-analysis inference for each exponentiated effect (i.e., ratio of expected SNP distances per specified change in covariate value). Posterior means and 95% quantile-based credible intervals are presented.

Effect	Estimate	95% Credible Interval
Distance Between Centroids (50 km)	1.03	(1.02, 1.05)
Same Centroid (Yes vs. No)	0.77	(0.68, 0.88)
Date of Diagnosis Distance (1/2 year)	1.01	(1.00, 1.02)
Age Difference (10 years)	1.00	(1.00, 1.02)
Age (10 years)	0.99	(0.98, 1.01)
Household Contacts (1 person)	1.00	(0.98, 1.01)
Sex:		
Mixed Pair vs. Both Female	0.95	(0.91, 1.00)
Both Male vs. Both Female	0.91	(0.83, 1.00)
Residence Location:		
Mixed Pair vs. Both Not Urban	1.02	(0.97, 1.08)
Both Urban vs. Both Not Urban	1.05	(0.95, 1.18)
Housing:		

Mixed Pair vs. Both Not Homeless	1.02	(0.89, 1.18)
Both Homeless vs. Both Not Homeless	1.07	(0.78, 1.44)
Working Status:		
Mixed Pair vs. Both Unemployed	1.03	(0.97, 1.12)
Both Employed vs. Both Unemployed	1.10	(0.95, 1.27)
Education:		
Mixed Pair vs. Both Secondary	0.98	(0.94, 1.02)
Both < Secondary vs. Both Secondary	0.97	(0.90, 1.04)

S6 Inference of person-to-person transmission events

We identified person-to-person transmission events between sampled hosts in large transmission clusters (⩾ ten cases, TreeCluster distance threshold 0.001 substitutions/site) by reconstructing transmission networks using TransPhylo.

For each large transmission cluster (⩾ ten cases), we ran TransPhylo on 50 random trees drawn from a posterior selection of 10,000 timed trees produced in BEAST2, discarding the first 50% as burn-in, for a total of 10^5 MCMC iterations. The parameter estimates for the offspring distribution, sampling density, and within-host coalescent rate were shared through multi-tree runs. For prior parameter values, the generation and sampling time prior parameters were represented by a gamma distribution with shape 1.3 and scale 3.33 (mean 4.3 years, SD 3.8 years), and 1.1 and 2.75 (mean 3.0 years, SD 2.9 years) respectively. While these prior distributions can have an impact on the resulting transmission inference, these values been used previously in *M. tuberculosis* transmission analyses and allow the majority of transmission events occur within 2-3 years of the transmitting host being infected, but also accounts for the potential for long periods of asymptomatic latency. The offspring distribution was a negative binomial distribution with parameters r = 1 (updated) and p = 0.5 (fixed), and the within-host coalescent rate was fixed at 100/365. The sampling density (proportion of sampled cases) was updated through iterations and drawn from a beta distribution with strong informative priors α = 20 and ß = 8, reflecting the high proportion of culture-positive cases captured in this study. The resulting transmission trees were assessed to determine person-to-person transmission events between sampled hosts with a posterior probability of ⩾0.5 (direct links between cases were found in more than half of the posterior transmission trees).

REFERENCE

1. Balaban M, Moshiri N, Mai U, Jia X, Mirarab S. TreeCluster: Clustering biological sequences using phylogenetic trees. PLoS One. 2019;14(8):e0221068. Epub 2019/08/23. doi: 10.1371/journal.pone.0221068. PubMed PMID: 31437182; PubMed Central PMCID: 6705769.
2. Bouckaert R, Vaughan TG, Barido-Sottani J, Duchene S, Fourment M, Gavryushkina A, et al. BEAST 2.5: An advanced software platform for Bayesian evolutionary analysis. PLoS Comput Biol. 2019;15(4):e1006650. Epub 2019/04/09. doi:

10.1371/journal.pcbi.1006650. PubMed PMID: 30958812; PubMed Central PMCID: 6472827.
3. Menardo F, Duchene S, Brites D, Gagneux S. The molecular clock of Mycobacterium tuberculosis. PLoS Pathog. 2019;15(9):e1008067. Epub 2019/09/13. doi: 10.1371/journal.ppat.1008067. PubMed PMID: 31513651; PubMed Central PMCID: 6759198.
4. Holt KE, McAdam P, Thai PVK, Thuong NTT, Ha DTM, Lan NN, et al. Frequent transmission of the Mycobacterium tuberculosis Beijing lineage and positive selection for the EsxW Beijing variant in Vietnam. Nat Genet. 2018;50(6):849-56. Epub 2018/05/23. doi: 10.1038/s41588-018-0117-9. PubMed PMID: 29785015; PubMed Central PMCID: 6143168.
