# Supplementary material for: Phylogeography and transmission of M. tuberculosis in Moldova: A prospective genomic analysis
Source: PLoS Med. 2022 Feb 22;19(2):e1003933. doi: 10.1371/journal.pmed.1003933 (PMC8903246; doi:10.1371/journal.pmed.1003933)
Supplement: Attachment — Submitted filename: Response letter_final.docx [file pmed.1003933.s004.docx]

**Response to comments from Editors:**

1)      Please revise your title according to PLOS Medicine's style. Your title must be nondeclarative and not a question. It should begin with main concept if possible. Please place the study design in the subtitle (i.e., after a colon). For example, “Phylogeography and transmission of M. tuberculosis in Moldova: A prospective genomic analysis” 

**Response: We have adopted your suggested new title: “Phylogeography and transmission of M. tuberculosis in Moldova: A prospective genomic analysis”. (page 1)**

2)      The Data Availability Statement (DAS) requires revision. If part of the data is not freely available, please include an appropriate contact (web or email address) for inquiries (this cannot be a study author/ co-author).

**Response: We have modified the DAS: The genomic data have been made available through GenBank (PRJNA736718). Additional data used in the analysis (with the exception of location data which cannot be provided because of the small number of participants at locations would allow linkage to individual participants), are provided as a csv in the Supplemental Material.**

3)      At this stage, we ask that you write a non-technical Author Summary. The Author Summary should immediately follow the Abstract in your revised manuscript. This text is subject to editorial change and should be distinct from the scientific abstract. The summary should be accessible to a wide audience that includes both scientists and non-scientists. Please see our author guidelines for more information: <https://nam12.safelinks.protection.outlook.com/?url=https%3A%2F%2Fjournals.plos.org%2Fplosmedicine%2Fs%2Frevising-your-manuscript%23loc-author-summary&amp;data=04%7C01%7Cchongguang.yang%40yale.edu%7C737dd18ee13b42f4c03b08d989870c65%7Cdd8cbebb21394df8b4114e3e87abeb5c%7C0%7C0%7C637692035395617911%7CUnknown%7CTWFpbGZsb3d8eyJWIjoiMC4wLjAwMDAiLCJQIjoiV2luMzIiLCJBTiI6Ik1haWwiLCJXVCI6Mn0%3D%7C1000&amp;sdata=7%2Fw4dj9WKinpRmKaOfntoyk0julVuGvK8PDZxfebdQo%3D&amp;reserved=0>.

**Response: We have added the summary to the main text.**

4)      Abstract:
a)      Please ensure that all numbers presented in the abstract are present and identical to numbers presented in the main manuscript text.

**Response: We have confirmed that the numbers in abstract the main text and appendix are consistent.**

b)      In the last sentence of the Abstract Methods and Findings section, please describe the main limitation(s) of the study's methodology.

**Response: Done. We have added text to clarify that the 2-year duration of the study and the possibility of TB cases missing from the analysis because of failure to diagnose or absence of culture-positivity are the main limitations of the analysis. (Abstract, page 18 and paragraph 1)**

5)      Did your study have a prospective protocol or analysis plan? Please state this (either way) early in the Methods section:
  a) If a prospective analysis plan (from your funding proposal, IRB or other ethics committee submission, study protocol, or other planning document written before analyzing the data) was used in designing the study, please include the relevant prospectively written document with your revised manuscript as a Supporting Information file to be published alongside your study and cite it in the Methods section. A legend for this file should be included at the end of your manuscript. 
b) If no such document exists, please make sure that the Methods section transparently describes when analyses were planned, and when/why any data-driven changes to analyses took place. 
c) In either case, changes in the analysis-- including those made in response to peer review comments-- should be identified as such in the Methods section of the paper, with rationale.

**Response: We had a prospective analysis plan before analyzing the data in this work which is included in the IRB of this study. The IRB document describing the analysis plan is uploaded as a supporting file along with this revision.**

6)      Please specify whether informed consent was written or oral.

**Response: Informed consent was provided in writing. We have added this to the methods section (page 5 paragraph 3)**

7)      Please ensure that the study is reported according to the STROBE guideline for observational studies, and include the completed STROBE checklist as Supporting Information. Please add the following statement, or similar, to the Methods: "This study is reported as per the Strengthening the Reporting of Observational Studies in Epidemiology (STROBE) guideline (S1 Checklist)." The STROBE guideline can be found here: <https://nam12.safelinks.protection.outlook.com/?url=http%3A%2F%2Fwww.equator-network.org%2Freporting-guidelines%2Fstrobe%2F&amp;data=04%7C01%7Cchongguang.yang%40yale.edu%7C737dd18ee13b42f4c03b08d989870c65%7Cdd8cbebb21394df8b4114e3e87abeb5c%7C0%7C0%7C637692035395617911%7CUnknown%7CTWFpbGZsb3d8eyJWIjoiMC4wLjAwMDAiLCJQIjoiV2luMzIiLCJBTiI6Ik1haWwiLCJXVCI6Mn0%3D%7C1000&amp;sdata=Za3NX8n7cKJMdS3EUISOj7HNbZhNKKyzqYdNNmv83sk%3D&amp;reserved=0>
**Response: we have uploaded the STROBE guideline in the submission system.**

8)      Your study is observational and therefore causality cannot be inferred. Please remove language that implies causality throughout the manuscript, such as “... the result of...” For example, you state in line 396-397 that “Our analysis reveals that this heterogeneity is the result of…” Please refer to associations instead.

**Response: We have changed the language throughout the manuscript to remove causal statements.**

9)      In the Methods and Results section, please consistently provide 95% CIs and p values for estimates in the main text and tables.

**Response: Done.**

10)     Figures 1-3, S4-S5:
a)      Please ensure that Figure 1 complies with our figure requirement: <https://nam12.safelinks.protection.outlook.com/?url=http%3A%2F%2Fjournals.plos.org%2Fplosmedicine%2Fs%2Ffigures&amp;data=04%7C01%7Cchongguang.yang%40yale.edu%7C737dd18ee13b42f4c03b08d989870c65%7Cdd8cbebb21394df8b4114e3e87abeb5c%7C0%7C0%7C637692035395617911%7CUnknown%7CTWFpbGZsb3d8eyJWIjoiMC4wLjAwMDAiLCJQIjoiV2luMzIiLCJBTiI6Ik1haWwiLCJXVCI6Mn0%3D%7C1000&amp;sdata=ZLrOULXGvMeA%2F%2BrRcjh6AmJN95SzUZ%2F%2FgABnDhAFv7g%3D&amp;reserved=0>.

**Response: We have modified the figures 1-4 following the journal requirements.**

b)      Please confirm that the appropriate usage rights apply to the use of this Figure 1. Please see our guidelines for map images: <https://nam12.safelinks.protection.outlook.com/?url=https%3A%2F%2Fjournals.plos.org%2Fplosmedicine%2Fs%2Ffigures%23loc-maps&amp;data=04%7C01%7Cchongguang.yang%40yale.edu%7C737dd18ee13b42f4c03b08d989870c65%7Cdd8cbebb21394df8b4114e3e87abeb5c%7C0%7C0%7C637692035395617911%7CUnknown%7CTWFpbGZsb3d8eyJWIjoiMC4wLjAwMDAiLCJQIjoiV2luMzIiLCJBTiI6Ik1haWwiLCJXVCI6Mn0%3D%7C1000&amp;sdata=8bmIueTs29YyvEN%2F9Rk7W1Z1Bj%2BEYhKRW17jopoBFR4%3D&amp;reserved=0>

**Response: We have confirm that the map of location of Moldova in Europe is adapted from Wikipedia and is under the “Public domain” license, which is in compatible with CC BY licensing. (reference link: https://en.wikipedia.org/wiki/File:Location_Moldova_Europe.png)**

11)     Please do not report P<0.01, instead report as P < 0.001.

**Response: We have corrected this.**

12)     Please remove the “Role of the funding source”, “Declaration of interests”, and “Data sharing” from the main text. This information will be published as metadata based on your responses to the submission form.

**Response: We have removed these three sections and filled the form in the submission system.**

13)     Please use PloS Medicine style in-text reference call outs noting the square brackets. For example "... countries [1,2]."

**Response: We have changed the reference format through the text.**

---------------------------------------------------------------------------------------

**Response to comments from the reviewers:**

Reviewer #1: General Comments
Yang and colleagues present the results of a prospective genomic analysis of all TB cases in the Republic of Moldova diagnosed in 2018 and 2019. The authors used next generation sequencing and phylogenetic methods to determine the proportion of cases attributable to multi-drug resistant M. tuberculosis (MDR-TB) variants and characterize their genetic clustering and spatial distribution. Finally, they identified independent individual and geographic predictors of recent transmission of MDR-TB. The authors conclude that the MDR-TB epidemic in Moldova is driven by local transmission and that there is an urgent need to apply comprehensive genomic surveillance to understand transmission and potentially to inform the design of public health interventions to contain the DR-TB epidemic.

Major Comments
This is a rigorously performed study that seeks to answer a provocative, if open-ended question, about what can be learned when demographic, spatiotemporal, and pathogen genomic data are integrated together at population level to provide a broad picture of an MDR-TB epidemic in a high-incidence country.

I have a few brief questions and comments about the methods, presentation, and interpretation, as well as a larger question about the place of this work in the literature and its implications.

1)      The authors mention the comprehensive sampling approach as a strength and note that some prior studies did not do this, including a recent study from Moldova. Is it possible to comment on how the inferences changed empirically with comprehensive sampling, and if so how? Are there any alternatives (e.g. stratified random samples) to comprehensive sampling that could improve feasibility without sacrificing rigor?

**Response: Previous studies on the molecular epidemiology of TB have highlighted issues associated with sampling biases when making inference on clustering and transmission (for example: Murray M, Alland D. Methodological problems in the molecular epidemiology of tuberculosis. Am J Epidemiol. 2002 Mar 15;155(6):565-71. doi: 10.1093/aje/155.6.565. PMID: 11882530; Murray M. Sampling bias in the molecular epidemiology of tuberculosis. Emerg**

**Infect Dis. 2002 Apr;8(4):363-9. doi: 10.3201/eid0804.000444. PMID: 11971768;**

**PMCID: PMC2730247; and Glynn JR, Vynnycky E, Fine PE. Influence of sampling on estimates of clustering and recent transmission of Mycobacterium tuberculosis derived from. DNA fingerprinting techniques. Am J Epidemiol. 1999 Feb 15;149(4):366-71. doi: 10.1093/oxfordjournals.aje.a009822. PMID: 10025480. Given the sensitivity of inference to the completeness of sampling, we do not think there are clear approaches for more efficient sampling that guard against these types of bias.**

2)      Reduced reagent volumes were one of the techniques the authors used to improve the affordability of comprehensive WGS. Should this not be highlighted as a strength or innovation of the study? Are the other learnings to improve feasibility that might inform replication of this study for research or practice?

**Response: We do not yet think there are major lessons to be learned at this point about our sequencing approaches which use reduced reagent volumes. There is a preprint ([https://www.biorxiv.org/content/10.1101/779215v1.full.pdf](https://www.biorxiv.org/content/10.1101/779215v1.full.pdf)) on the potential of approaches for improving affordability of sequencing in LMIC, but we do not see this as the major obstacle toward improving local capacity for sequencing, and have chosen not to emphasize this in this manuscript.**

3)      Can the authors state somewhere why was Moldova chosen as a location for this study? Can the authors comment on the possible generalizability of these findings to other countries with DR-TB epidemics, in the region and possibly beyond? Presumably every country has its unique features, but are there particular characteristics of Moldova (beyond its small size) or of its MDR-TB epidemic that made it more suitable for this project than others?

**Response: Moldova was chosen for this study for several reasons. First, Moldova is on the WHO list of high MDR TB burden countries with one of the highest proportions of MDR among TB cases worldwide, making understanding the dynamics of MDR in this setting of great importance (page 5 and paragraph 1). Second, our research group has developed a strong collaboration with Moldova over the past decade, making it possible to conduct this type of prospective country-wide study. Third, Moldova has a strong national tuberculosis laboratory system; this was key for this study since samples from all TB suspects were tested by solid and liquid culture for mycobacterial growth. This ensured that we were able to capture all bacteriologically positive cases in the country, which, to our knowledge, is unique for high MDR-TB prevalence countries. While the role of genomic surveillance for informing TB interventions in high-burden settings remains incompletely explored, we believe that, in general, this study provides an important example of how such information may be used to understand the complex epidemiology of MDR-TB in a high incidence country.**

4)      The authors present a detailed analysis of pathogen and demographic factors that may drive transmission, but there is also very limited clinical information about host factors. Could the authors speculate to what extent could HIV, or other host factors that also cluster in individuals, might act as confounders of the observed associations? Should this type of clinical information be captured in a comprehensive surveillance system?

**Response: As part of this study, we utilized only data collected at the time of TB diagnosis within the TB surveillance system (which does not include a detailed medical history). Known HIV co-infection at the time of TB diagnosis is recorded, but the prevalence of HIV coinfection among TB patients is small in Moldova, constituting approximately 5% of all TB cases, so this is unlikely to be a major determinant of the distribution of TB lineages reported in this study.**

5)      Can the authors comment on the potential effects of excluding polyclonal infections? I understand that this is technically necessary, but it would help to hear the implications for the overall analysis. For example, to the extent that mixed infections reflect greater transmission, could the overall estimates of recent and local transmission be underestimates?

**Response: We removed the mixed/polyclonal infection samples from the analysis due to difficulties in producing well-resolved phylogenies, though we found evidence for homogeneous and heterogenous drug resistance mutations in these samples, including MDR-conferring variation found at a similar proportion to the remaining study population. The exclusion of mixed samples in the main clustering analysis could lead to an underestimation of transmission as the mixed or polyclonal infections often reflect greater transmission force in the population, though it may be difficult to determine the separated genomic patterns from the mixed one. Further analysis would be required to understand the role of mixed TB infection in transmission and drug resistance TB within Moldova. We have added a section in the discussion to address this as a limitation. (page 18 paragraph 1)**

6)      Some additional explanation to help contextualize these findings would help highlight its importance to general audiences. Specifically, the application of TB molecular epidemiology methods to improve global TB control has been somewhat disappointing. To what extent are the two innovations described here, whole genome sequencing and comprehensive surveillance, well-positioned to overcome some of the limitations of prior approaches, and, if adopted, better inform design of interventions to improve TB control and elimination? What are the next steps, scientifically, to move from the detailed understanding provided in this paper to such interventions?

**Response: We agree that this is an important question – to what extent does a detailed** **understanding of local transmission afforded by WGS in the context of comprehensive surveillance allow for better targeting of TB interventions? We have highlighted for readers the importance of this question and the future directions of this work by adding the following sentence to the end of the Discussion (page 18 and paragraph 2):**

**“We must next investigate whether this improved understanding of local transmission can inform the design of more effective and efficient interventions, a question which remains unanswered at this time.**”

Minor Comments:
Results
Could the authors report the prevalence of pre-XDR and XDR isolates? Figure 2 suggests that the number of rrs and rlsb mutations may be large but the number of gyrA mutations appears small.

**Response: We added the number of pre-XDR and XDR in the results section. (page 11 and paragraph 1)**

Figures and Tables
Figure 1. Please state the number of participants shown in the figure, as well as the total number of districts and regions. Could you elaborate on "The scheme of color represents the distribution of notified incidence and localities with missing population data are in gray." Most of the figure(s) are gray - did you really mean missing denominators, or missing cases (as in no cases reported?), which is what the legend implies?

**Response: Thank you for these comments. We added the total number of participants and the number of regions in figure 1’s caption (page 10). The gray color in incidence panel represents the missing denominators, this information is now included in the revised figure legend (page 10). We also increased the figure resolution and changed the color scheme from gray to dark gray, so the color difference is clearer.**

A flow diagram, as specified by STROBE guidelines, is needed to aggregate in one figure all data on patient inclusion in and exclusion from analyses. This could appear in the Supplement but should be referenced in the main text. The inclusion of a high proportion of the eligible population is actually a strength of the study.

**Response: We have added a flow diagram to the supplement and refermced it in the text. (page 1, figure S1 in appendix).**

There appears to be a discrepancy in the number of polyclonal infections reported in the text (n=386) and the number of mixed infections in the supplement (n=403) - I assume that these should be the same.

**Response: Thanks. We have corrected this mismatch. The correct number was 386.**

The legend and other aspects of Table S4 on Page S18 printed partly outside the margins.

**Response: we have reduced the size of Table S4 (S7 in revision) and made all related aspects within the printable margin.**


Reviewer #2: General comments
The manuscript is clearly described and provide additional insight into the MDR-TB transmitted by heterogeneity is the result of multiple overlapping epidemic in Moldova. 
The subject of the manuscript is of importance as 3 main clades of M.tb transmission have been well addressed in the study,the finding could be useful for public health response to address this issue.

Major comments
1.the SNP threshold is crucial to define the cluster and explore the transmission, given in the high TB epidemic setting, the genetic evolution might be varied under the different selection pressures, so for the genomic similarity, it will be reasonable if the author describe why the SNP thresholds of 40 and 20 were chosen, rather than 12 or 18.  As we know, under these thresholds and the cluster rates tend to be decreased accordingly. the author had better make analysis and add relevant content in the paper.

**Response: We have conducted additional analyses and added the relevant results in the appendix. We used phylogenetic clustering analysis and chose the patristic distance (0.001 and 0.0005 thresholds or 40 and 20 SNPs, respectively), which is the maximum distance within the cluster; thus, we were able to capture the clonal transmission of the strains formed in one cluster (e.g., both recent and remote transmission). We emphasized this in the revised manuscript (page 6 and paragraph 3).**

**Perhaps more relevant to the concern raised, we have also now presented the median pairwise SNP distance within the cluster, and in 0.001 cutoff clusters, the median SNP distance was 14 SNPs (IQR 10-18 SNPs). We then conducted the additional analysis for 0.0005 cutoff clusters as requested by this comment, and the median SNP distance within a cluster was 9 SNPs (IQR 7-12 SNPs). We added these results to the main text (page 12 and paragraph 3) and supplemental materials (page 7-8, figure S4 in the appendix). These between host SNPs are quite consistent with other work using pairwise SNP distances to define recent transmission.**

Minor comments
1.Within this manuscript the authors have looked at whole picture of Moldova,  in this study, the author have collected all culture-positive isolate in Moldova, however, infection and development of TB  should be pointed out as the limitation and bias of the un- recovered strain and missing case.

**Response: We agree that despite the collection of all culture-positive samples, we still cannot assure full capture as there are undiagnosed TB cases, TB cases that were not culture-positive, and we are limited by the relatively short time of the study. We have emphasized these limitations in the revised text (page 18 and paragraph 1; please see reponsnes to other reviewers as well).**

2. inference of direct transmission  it seems solid analysis, however, in this study,  as to chronic infectious disease with relatively unclear exposure history, how about the in-direct transmission.

**Response: We are not sure we understand this comment perfectly, but we have removed the use of the term “direct transmission” from the manuscript to avoid confusion. We agree that the chronic nature of TB disease and natural history, makes inference on transmission from a short study difficult. We have stressed this limitation, but we believe our insights that recent local transmission is dominanting TB epidemiology in this setting are robust to this limitation, in that a short study is likely to underestimate (rather than overestimate) transmission due to cases within transmission chains being missing from the study.**

Reviewer #3: The study is relevant to understanding the distribution and dynamics of TB, with important approaches in phylogenetic analysis and transmission cluster identification as well as in genomic analysis and phylogeny reconstruction. The methodology used is consistent with the objective of the study, which was approved by the Ethics Committee and has a consenting participant enrolment.
The results showed in detail the Map of culture-confirmed TB patients in Moldova, the phylogenetic distribution of resistance-related genotypes, the prevalence of drug resistance genotypes, the transmission of drug-resistant M. tuberculosis, and the estimate of M. tuberculosis effective population size for the three major clades over time, which collaborates to intervene in the dynamics of disease transmission. The tables present relevant data (demographic and clinical characteristics of study participants/pooled bayesian meta-analysis inference for each effect on the relative risk scale) and the figures clearly present the results of the study.
The discussion was carried out with a scientific basis and with a description of the strength and limitation of the study, which corroborates for further studies to be carried out and provides an important example of how such information may be used to understand the complex epidemiology of MDR-TB in a high incidence country.
I congratulate the authors for their initiative in this study and their scientific collaboration in TB control.

**Response: Thank you for these kind words!**


Reviewer #4: The authors of "Phylogeography and transmission of M. tuberculosis in Moldova" have done a great job at elucidating the phylogenetic intricacies of TB spread in Moldova, using robust methodologies for phylogeny inference. The biggest strength of this study is that it includes all TB-positive samples from the whole country of Moldova, and hence provides a good overview of the situation concerning tuberculosis in the country. In general, this study is well designed, implemented, and written. However, the huge amount of whole genome sequence data generated by this study could yield more informative results if analyzed a bit further. Kindly find bellow my suggestions.

A- On line 132 the authors state that in silico drug resistance prediction was carried out using TB-Profiler. Other studies exploring the in silico drug resistance genotypes of TB have noted that the use of more than one software to predict these genotypes would yield complementary results. Although I share the methodological viewpoint of the authors that currently TB-Profiler holds the throne for in silico drug resistance genotyping of TB with its huge database and robust methodology, I am curious to know whether the authors tested a few other bioinformatic tools such as Mykrobe or KvarQ. 
Additionally, a study of this scale would benefit from the implementation of a newly developed methodology that reports to have a slightly higher sensitivity than TB-Profiler. Although still in draft form, the tool GenTB reportedly has 3.2% higher sensitivity compared to TB-Profiler in detecting resistance conferring mutations for the nine second-line anti-TB drugs. Seeing as how this could mean that up to 71 samples from this study could have their resistance profiles altered, I suggest that the authors implement the approach outlined in [<https://nam12.safelinks.protection.outlook.com/?url=https%3A%2F%2Fgithub.com%2Ffarhat-lab%2Fgentb-site&amp;data=04%7C01%7Cchongguang.yang%40yale.edu%7C737dd18ee13b42f4c03b08d989870c65%7Cdd8cbebb21394df8b4114e3e87abeb5c%7C0%7C0%7C637692035395627897%7CUnknown%7CTWFpbGZsb3d8eyJWIjoiMC4wLjAwMDAiLCJQIjoiV2luMzIiLCJBTiI6Ik1haWwiLCJXVCI6Mn0%3D%7C1000&amp;sdata=mqq1%2BZgQTzTXUpG8D78mNFyQInrS78EIZpraumDxDeg%3D&amp;reserved=0>] and report any changes to their in silico drug resistance prediction results.

**Response: We randomly selected 10% (n=183) of our samples and tested our DR prediction using the weblink (i.e. the genTB online tool). After excluding one sample with low reads, the prediction of MDR-TB (INH and RIF resistance) was 100% match between the online genTB tool and TBprofiler we used in the manuscript. The genTB tool, however, missed a number of important second-line resistance predictions; for example, it missed almost all the kanamycin resistance predictions which we double checked our variants outputs. We have added these results to the Supplemental Appendix (table S3). As it states on its webpage that this is a beta version and the online processing is not stable, we do not think it is appropriate to do a formal comparison between TBprofiler and the current form of genTB.**

B- On line 127, and in more detail in the supplementary data, the authors state that samples exhibiting heterogeneity were discarded from the analysis. Although their aim to minimise the discordance between genomic variation phylogenetic relatedness clarifies as to why 18% of their samples were removed from the phylogenetic analyses, nevertheless, I believe that there is potential in analysing these 403 heterogeneous samples. 
Other publications have shown that polyclonal TB infections are of clinical interest as highlighted in (<https://nam12.safelinks.protection.outlook.com/?url=https%3A%2F%2Fdx.doi.org%2F10.3201%252Feid2311.170077&amp;data=04%7C01%7Cchongguang.yang%40yale.edu%7C737dd18ee13b42f4c03b08d989870c65%7Cdd8cbebb21394df8b4114e3e87abeb5c%7C0%7C0%7C637692035395627897%7CUnknown%7CTWFpbGZsb3d8eyJWIjoiMC4wLjAwMDAiLCJQIjoiV2luMzIiLCJBTiI6Ik1haWwiLCJXVCI6Mn0%3D%7C1000&amp;sdata=rlFFRr2zM3Fb9Uzw89bMK7sJWYmtwdwX1Jh57xQVrAQ%3D&amp;reserved=0>) this paper also senior authored by Dr. Cohen. Therefore, I suggest that these 403 samples be checked for the presence of mutations encoding drug resistance, and reported in a table format without a phylogeny simply for the added value of this analysis to report the MDR status of polyclonal infections in Moldova. Consequently, there should be a brief discussion on the importance of polyclonal TB infections and their clinical implications 
<https://nam12.safelinks.protection.outlook.com/?url=https%3A%2F%2Fwww.nature.com%2Farticles%2Fsrep41410&amp;data=04%7C01%7Cchongguang.yang%40yale.edu%7C737dd18ee13b42f4c03b08d989870c65%7Cdd8cbebb21394df8b4114e3e87abeb5c%7C0%7C0%7C637692035395627897%7CUnknown%7CTWFpbGZsb3d8eyJWIjoiMC4wLjAwMDAiLCJQIjoiV2luMzIiLCJBTiI6Ik1haWwiLCJXVCI6Mn0%3D%7C1000&amp;sdata=3GG19xBRj5ityGLD91qRwK11xlnu3ucmNeJVOblu7f0%3D&amp;reserved=0>
<https://nam12.safelinks.protection.outlook.com/?url=https%3A%2F%2Fjournals.plos.org%2Fplosone%2Farticle%3Fid%3D10.1371%2Fjournal.pone.0237345&amp;data=04%7C01%7Cchongguang.yang%40yale.edu%7C737dd18ee13b42f4c03b08d989870c65%7Cdd8cbebb21394df8b4114e3e87abeb5c%7C0%7C0%7C637692035395627897%7CUnknown%7CTWFpbGZsb3d8eyJWIjoiMC4wLjAwMDAiLCJQIjoiV2luMzIiLCJBTiI6Ik1haWwiLCJXVCI6Mn0%3D%7C1000&amp;sdata=KvXPNb6Eg%2BM2sWiq9vhCnTgIuP%2BVbuW2HbiTl%2BttNnA%3D&amp;reserved=0>
<https://nam12.safelinks.protection.outlook.com/?url=https%3A%2F%2Fwww.sciencedirect.com%2Fscience%2Farticle%2Fpii%2FS1472979217302251&amp;data=04%7C01%7Cchongguang.yang%40yale.edu%7C737dd18ee13b42f4c03b08d989870c65%7Cdd8cbebb21394df8b4114e3e87abeb5c%7C0%7C0%7C637692035395627897%7CUnknown%7CTWFpbGZsb3d8eyJWIjoiMC4wLjAwMDAiLCJQIjoiV2luMzIiLCJBTiI6Ik1haWwiLCJXVCI6Mn0%3D%7C1000&amp;sdata=T%2BTABvMaOGT%2F4LpyUz6d8vjAYWTKNscmvHowffcbTxU%3D&amp;reserved=0>
<https://nam12.safelinks.protection.outlook.com/?url=https%3A%2F%2Fwww.sciencedirect.com%2Fscience%2Farticle%2Fpii%2FS1472979221000706&amp;data=04%7C01%7Cchongguang.yang%40yale.edu%7C737dd18ee13b42f4c03b08d989870c65%7Cdd8cbebb21394df8b4114e3e87abeb5c%7C0%7C0%7C637692035395627897%7CUnknown%7CTWFpbGZsb3d8eyJWIjoiMC4wLjAwMDAiLCJQIjoiV2luMzIiLCJBTiI6Ik1haWwiLCJXVCI6Mn0%3D%7C1000&amp;sdata=X%2FPLJg1T7WVLIXENUOLNgSqR8Mn8MVndx%2B%2FXFSxNrIY%3D&amp;reserved=0>

I hope that these suggestions aid in improving your already well-drafted manuscript

**Response: Thank you for this suggestion. We conducted further analysis on these polyclonal samples and have incorporated these findings into the revised manuscript. Specifically, we investigated drug resistant mutations in these polyclonal samples and made two additional tables listing the lineage classification and the number of drug resistant samples with heterogenous sites. (Table S1 and S2 in supplemental appendix)**


Reviewer #5: Alex McConnachie, Statistical Review

The paper by Yang and colleagues looks at the genetics of tuberculosis cases in Moldova in 2018/19. Much of the paper is beyond my area of expertise, but I can comment on the spatial/genetic distance analysis described from line 162 onwards.

This analysis takes each pair of cases within a cluster as the unit of analysis, and treats the patristic distance between them as the outcome. Again, this is not my field, but I believe this is a measure of the "genetic distance" between two cases. The predictors are measures of within-pair differences, in space, time, or demographics, and some individual demographic measures.

The model assumes that the log-transformed patristic distance is a linear function of the predictor variables, with Normally-distributed, independent errors. The authors do not give any indication of whether these assumptions about the error part of the model are reasonable. I would be particularly concerned about the independence assumption, since the outcomes are derived from pairs of cases. I would expect this to lead to some in-built correlation between residuals. In general, failure to account for correlations between residuals will tend to result in overly-precise estimates of associations.

The results of these analyses are combined across clusters using a Bayesian meta-analysis, which is good. However, the associations are reported as relative risks, which seems wrong, since the outcome is continuous. I am never quite sure of the best term to use, but the exponentiated regression coefficients could perhaps be described as ratios of geometric means associated with a given change in a predictor, but if the authors can think of a better descriptor, that would be fine.

**Response: We thank Dr. McConnachie for these incisive comments -- we have recently been working on this topic and have now developed novel hierarchical Bayesian methods to address potential correlation in paired genetic relatedness data which we have posted as a pre-print and submitted to a statistical journal. (reference:** [**https://arxiv.org/abs/2109.14003**](https://arxiv.org/abs/2109.14003)**). In the revised text we re-analyzed the data using these methods and quantify the association between the genetic and spatial distances for unique pairs of cases, adjusting for other pair- and individual-level features and multiple sources of correlation in the data. The pooled results that we present did not change dramatically. We modified the results in the table and text in the results section accordingly (page 15 and paragraph 2, table 2 in the main text and page 27 and table S8 in the appendix).**

**We also removed the term “relative risk” as we are working with log patristic distances, and the exp(beta) represents the % change in the expected value of Y given a one unit increase in in a specific covariate value. We make this clear in the caption for Table 2.**

Similar comments could be made about the Poisson version of this analysis reported in the appendix. Are the errors really independent? Is the Poisson distribution assumption (mean = variance) reasonable? Is "relative risk" a sensible term to describe the estimated associations?

**Response: Thank you for these comments. We also re-analyzed these data using the new methodology and update the results. In this analyses we also use the negative binomial distribution to account for potential overdispersion. Finally, we remove the term “relative risk” from the description and more specifically define the results in terms of ratios of expected values based on changes in covariates.**

One final comment - logistic regression is used to look at factors associated with a case being in a "large cluster", but this is not described in the methods section.

**Response: Thank you for pointing out this oversight. We have added a description to the Method section in the appendix text (page 10 paragraph 1 in supplemental appendix).**
