# Supplementary material for: Phylogeography and transmission of M. tuberculosis in Moldova: A prospective genomic analysis
Source: PLoS Med. 2022 Feb 22;19(2):e1003933. doi: 10.1371/journal.pmed.1003933 (PMC8903246; doi:10.1371/journal.pmed.1003933)
Supplement: Attachment — Submitted filename: response_tc.docx [file pmed.1003933.s005.docx]

Requests from Editors:

1.      Please provide a web link to access GenBank data (PRJNA736718)

**Response:** we have added the full data link (https://www.ncbi.nlm.nih.gov/bioproject/PRJNA736718) to the data sharing box in the submission system.

2.      Author summary – Regarding the content under “What did the researchers do and find?” please trim it down to 3 or 4 bullet points.

**Response:** Thanks. We have trimmed this part down to 4 bullet points:

**What did the researchers do and find?**

- We prospectively enrolled all individuals with incident culture-positive TB from the Republic of Moldova, a high MDR-TB burden setting, between January 2018 and December 2019 and sequenced a diagnostic *M. tuberculosis* isolate from each individual.
- We found that that nearly all extant MDR-TB in Moldova is likely the result of recent transmission and that MDR is highly concentrated within two *M. tuberculosis* lineages (Beijing and Ural).
- Phylogeographic analyses revealed geographically distinct patterns of transmission for the Beijing MDR strains, which were predominantly localized within the Transnistrian region to the east of the country, while Ural MDR strains were less geographically restricted.
- Each putative MDR-TB transmission cluster had distinct second-line drugs resistance-conferring mutations. Population genetic analyses revealed both long periods of local population expansion as well as more recent introduction of specific MDR-TB strains into the country.

3.      Your study is observational and therefore causality cannot be inferred. Please remove language that implies causality throughout the manuscript abstract, summary, main text and conclusions, such as, “is fueled by …” Please refer to associations, e.g., “…is associated with …” or similar

**Response:** we have read through the manuscript and corrected the language that implies causality.

4.      The terms gender and sex are not interchangeable (as discussed in [https://nam12.safelinks.protection.outlook.com/?url=http%3A%2F%2Fwww.who.int%2Fgender%2Fwhatisgender%2Fen%2F&amp;data=04%7C01%7Cchongguang.yang%40yale.edu%7C81e439177fe34e1f6a8208d9d640cb52%7Cdd8cbebb21394df8b4114e3e87abeb5c%7C0%7C0%7C637776396063729431%7CUnknown%7CTWFpbGZsb3d8eyJWIjoiMC4wLjAwMDAiLCJQIjoiV2luMzIiLCJBTiI6Ik1haWwiLCJXVCI6Mn0%3D%7C3000&amp;sdata=lDAv6EuKYq3Fat3uq1Qgp89uQPxuGyFSpPm8XLN%2BxHw%3D&amp;reserved=0](https://nam12.safelinks.protection.outlook.com/?url=http%3A%2F%2Fwww.who.int%2Fgender%2Fwhatisgender%2Fen%2F&data=04%7C01%7Cchongguang.yang%40yale.edu%7C3d8c0292f2b64a3b923608d9d69a5066%7Cdd8cbebb21394df8b4114e3e87abeb5c%7C0%7C0%7C637776780525580519%7CUnknown%7CTWFpbGZsb3d8eyJWIjoiMC4wLjAwMDAiLCJQIjoiV2luMzIiLCJBTiI6Ik1haWwiLCJXVCI6Mn0%3D%7C3000&sdata=RMVeVYuGMLFB0SueBlD5hi4zelHug%2FkFd4LDA9%2BB4L8%3D&reserved=0) ); please use the appropriate term

**Response:** We replaced the “sex” with “gender” through the manuscript and the appendix materials.

5.      Please temper claims of primacy of results (e.g., the first study to…) by stating, "to our knowledge" or something similar.

**Response:** we added the “To our knowledge” to temper the primacy claim in the Author’s summary.

6.      Thank you for providing your STROBE checklist. Please replace the page numbers with paragraph numbers per section (e.g. "Methods, paragraph 1"), since the page numbers of the final published paper may be different from the page numbers in the current manuscript.

**Response:** Thanks for this suggestion. We have updated the STROBE checklist.

7.      Please provide the meaning of abbreviations used in tables and figures e.g. yr, IQR, SD, MDR. Please provide this in the footnotes.

**Response:** we added the relevant abbreviations’ meaning in each tables and figures.


Comments from Reviewers:

Reviewer #1: The Authors have addressed my previous questions and comments in their responses. I offer my congratulations on their work.

**Response:** Thank you for your previous review comments.

Reviewer #2: The author have asked and modified manuscript according to the comments, i feel comfortable about this version.

**Response:** Thank you for your previous review comments.

Reviewer #4: The authors have worked diligently to address the suggestions put forth by the other reviewers and myself. I have no further comments on this polished version of the manuscript

**Response:** Thank you for your previous review comments.


Reviewer #5: Alex McConnachie, Statistical Review

I thank the authors for their responses to my original points, which were more than satisfactory. I am sorry that they had to do all that extra work to fit a model that made little difference to the final results!

I noticed a possible typo. On line 219, just before the regression equation, the sentence ends "...and other covariates such as" which sounds as if something is missing.

I have no further comments.

**Response**: we appreciated your suggestion on the statistic analyses. We added the name of covariates into the text (which were the same as presented in table 2).
