# Supplementary material for: Phylogeography and transmission of M. tuberculosis in Moldova: A prospective genomic analysis
Source: PLoS Med. 2022 Feb 22;19(2):e1003933. doi: 10.1371/journal.pmed.1003933 (PMC8903246; doi:10.1371/journal.pmed.1003933)
Supplement: Attachment — Submitted filename: response letter.docx [file pmed.1003933.s006.docx]

Requests from Editors:

------------------------------------------------------------

1. Please replace the term "gender" with "sex." PLOS Medicine style is to use biological "sex" rather than "gender", where appropriate. Apologies for the confusion caused with the terms used.

Response: Apologies for the confusion. We now replaced the “gender” with “sex” through the manuscript.
